# Venom duct origins of prey capture and defensive conotoxins in piscivorous *Conus striatus*

**DOI:** 10.1038/s41598-021-91919-4

**Published:** 2021-06-24

**Authors:** S. W. A. Himaya, Ai-Hua Jin, Brett Hamilton, Subash K. Rai, Paul Alewood, Richard J. Lewis

**Affiliations:** 1 Institute for Molecular Bioscience, The University of Queensland, Saint Lucia, QLD 4072 Australia; 2 Centre for Microscopy and Microanalysis, The University of Queensland, Saint Lucia, QLD 4072 Australia; 3 Present Address: Genome Innovation Hub, The University of Queensland, Saint Lucia, QLD 4072 Australia

**Keywords:** Peptides, Proteome informatics, Mass spectrometry

## Abstract

The venom duct origins of predatory and defensive venoms has not been studied for hook-and-line fish hunting cone snails despite the pharmacological importance of their venoms. To better understand the biochemistry and evolution of injected predatory and defensive venoms, we compared distal, central and proximal venom duct sections across three specimens of *C. striatus* (*Pionoconus*) using proteomic and transcriptomic approaches. A total of 370 conotoxin precursors were identified from the whole venom duct transcriptome. Milked defensive venom was enriched with a potent cocktail of proximally expressed inhibitory α-, ω- and μ-conotoxins compared to milked predatory venom. In contrast, excitatory κA-conotoxins dominated both the predatory and defensive venoms despite their distal expression, suggesting this class of conotoxin can be selectively expressed from the same duct segment in response to either a predatory or defensive stimuli. Given the high abundance of κA-conotoxins in the *Pionoconus* clade, we hypothesise that the κA-conotoxins have evolved through adaptive evolution following their repurposing from ancestral inhibitory A superfamily conotoxins to facilitate the dietary shift to fish hunting and species radiation in this clade.

## Introduction

Cone snails are predatory marine gastropods that have evolved one of the most sophisticated envenomation strategies known, supporting their explosive radiation into over 850 species^1^. *Conus* venoms typically contain thousands of mostly disulfide-rich and highly structured peptides called conotoxins that target a wide range of neuromuscular receptors, ion channels and transporters^2,3^ to facilitate prey capture and for defence against predators^4–7^. Cone snails typically prey on worms (vermivorous), other molluscs (molluscivorous) or fish (piscivorous), with the predatory venoms of fish hunters evolved to target vertebrate receptors and ion channels.

Defensive venoms in worm hunting species appear to have facilitated the dietary shift to fish hunting in cone snails^4,8^. Currently 8 subgenera (clades) of cone snails are classified as fish hunters (*Pionoconus, Chelyconus, Gastridium, Textilla*, *Phasmoconus, Embrikena, Alfonsoconus and Asprella*) of the 68 identified clades, although direct fish hunting observations are missing in the *Embrikena, Alfonsoconus and Asprella* clades^9–11^. Fish hunting is proposed to have evolved from ancestral worm hunting cone snail species^4,12,13^ through the repurposing of defensive venom peptides^4^. Among these fish hunters, the *Pionoconus* clade is widely distributed, accounting 40 known fish hunting species^11^. Venoms of only a few species of *Pionoconus* clade (*C. magus*, *C. catus*, *C. consors* and *C. striatus*) have been studied extensively due to their medical potential^14–17^. These include the first marine drug ω-conotoxin MVIIA found in *C. magus*^18^, the related ω-conotoxin CVID from *C. catus* that showed efficacy in clinical trials^19^, and the potentially more selective CVIE and CVIF that have been investigated pre-clinically^20^.

The venom of cone snails is highly variable both between and within species^16,21–25^ as well as spatially along the venom duct^4,9,26^. Spatial differentiation in the venom gland has been shown to correlate with functionally distinct predatory and defensive venoms for the net feeding fish hunter *C. geographus*^4^. However, differentiation of defensive and predatory venoms in hook-and-line fish hunting clades and their venom duct origins has not been investigated. In this study, we used a combination of proteomics and transcriptomic approaches to decode the venom profiles of *C. striatus* from the *Pionoconus* clade. Characterisation of conotoxins between three individuals and their trisected venom ducts revealed differential expression amongst the 25 known superfamilies identified. The κA-conotoxins of the A superfamily and conkunitzins dominated the distal venom duct, while α-like conotoxins of the A superfamily, ω- and δ-like conotoxins of the O1 superfamily, μ-like conotoxins of the M superfamily, contryphans and con-ikot-ikots dominated the proximal venom duct. Analysis of the milked predatory and defensive venoms revealed that the distally dominant κA-conotoxins were expressed in response to both predatory and defensive stimuli. Findings of this study, together with previously published reports, suggest κA-conotoxins might be the key evolutionary drivers of successful radiation in *Pionoconus* clade cone snails.

## Results and discussion

### Transcriptomics reveals *C. striatus* venom complexity

Nine transcriptomes from trisected venom ducts (~ 4 cm distal, central and proximal sections) of three adult specimens (A, B and C) of *C. striatus* were obtained using NextSeq 300 cycle (2 × 150 bp) high output Illumina run (Table 1). Although this platform facilitates deep sequence analysis with a significantly low error rate (~ 0.1%), the relatively short read lengths (150 bp paired end) makes de novo transcript assembly an essential step to obtain full conotoxin transcript sequences (> 400 bp). A rationally designed in-house optimised *de-novo* assembly method using Trinity platform with conotoxin precursor (complete conotoxin transcript) annotation was used to unravel the *C. striatus* venom repertoire (supplementary Table S1). In comparison to default Trinity assembly parameters, the optimised method applied two kmers of sizes of 19 and 31 to capture both low and highly expressed transcripts, with SNP transcripts maintained and redundant transcripts removed during assembly. These parameters improved the capture of transcriptomic messiness^27^ and identified superfamilies and unique transcripts that were missed using the default parameters of the Trinity platform (supplementary Table S2).

**Table 1:** Integration of the transcriptome and proteomic data using ProteinPilot tool. Underlined sequences are matched to 99% confidence (list of peptide fragments are shown in supplementary table S8) to the proteomic data obtained from the three venom duct sections of *C. striatus.* MS/MS fragments obtained from reduced-alkylated and reduced-alkylated-trypsin digested extracted venom from three venom duct sections of specimens A, B and C of *C. striatus* were used to map their presence in the proteome.

Sequence ID	Superfamily	Mature sequence*	Distal	Central	Proximal
STR1_SI	A	ICCNPACGPKYSC	✓	✓	✓
STR14_Sm1.2	A	NGCCRNPACESHRC		✓	✓
STR17_SII	A	GCCCNPACGPNYGCGTSCS	✓	✓	✓
STR18	A	APALVVTATTNCCGYTGPACHPCLCTQTC	✓		
STR19	A	QKELVPSVITTCCGYDPGTMCPPCRCDNSCKPKPKK	✓		
STR20_SIVB	A	QKELVPSVITTCCGYDPGTMCPPCRCTNSC	✓		
STR21_SIVA	A	QKSLVPSVITTCCGYDPGTMCPPCRCTNSC	✓	✓	
STR22	A	QKSLVPSVITTCCGYDPGTMCPPCRCTNSCPKKPKKP	✓		
STR24_Sx4.1	A	QKSLVPSVITTCCGYDPGTMCPPCRCTNSC	✓		
STR25	A	QKSLVPSVITTCCGYDPGTMCPPCRCTNSCKTKPKK	✓		
STR26	A	QKELVPSVITTCCGYDPGTMCPPCRCTNSCKTKPKK	✓		
STR34	B2	KQHSQFNADENKAAFDSEDSLGNFMDFLHNEKGDKLPFANVDSAATDLGQFQPSAENEDGKFRFFDRQQ	✓	✓	✓
STR36	B2	KQHSQFNADENKAAFDSEDSLGNFMDFLHNEKGDKVPFANVDSAATDLGQFQPSAENEDGKFRFFDRQQ	✓	✓	✓
STR37	B2	KQHSQFNADENKAAFDSEDSLGNFMDFLHNEKGDKVPFANVDSAATDLGQFQPSAENEDGKFRFFDRQQ	✓		
STR39	B2	KQHSQFNADENKAAFDSEDSLGNFMDFLHNEKGDKLPFANVDSAAADLGQFQPSAENEDGKFRFFDRQQ		✓	
STR41	Con-ikot-ikot	SGPADCCRMKECCTDRVNECLQRYSGREDKFVSTCYQEATLTCGSFNEIVGCCYGYQMCMIRVVKPNSLSGAHEACKTVSCGNPCA		✓	
STR42_Con-ikot-ikot_SI	Con-ikot-ikot	SGPADCCRMKECCTDRVNECLQRYSGREDKFVSFCYQEATVTCGSFNEIVGCCYGYQMCMIRVVKPNSLSGAHEACKTVSCGNPCA	✓	✓	✓
STR52	Conkunitzin	DRPSLCDLPADSGSGTKAEKRIYYNSARKQCLRFDYTGQGGNENNFRRTYDCQRTCLYT	✓		
STR53	Conkunitzin	PSYCNLPADSGSGTKPEQRIYYNSAKKQCVTFTYNGKGGNGNNFSRTNDCRQTCQYPA	✓		
STR54	Conkunitzin	PSYCNLPADSGSGTKPEQRIYYNSAKKQCVTFTYNGKGGNGNNFSRTNDCRQTCQYPLYACISGCRCET	✓		
STR55_Conkunitzin-S2	Conkunitzin	ARPKDRPSYCNLPADSGSGTKPEQRIYYNSAKKQCVTFTYNGKGGNGNNFSRTNDCRQTCQYPV	✓	✓	
STR56_Conkunitzin-S1	Conkunitzin	KDRPSLCDLPADSGSGTKAEKRIYYNSARKQCLRFDYTGQGGNENNFRRTYDCQRTCLYT	✓	✓	
STR66	conopressin	CIIRNCPRGGKRDVDETHLTMPCMCCSFRQCGAEYLLWSWRMGNGDRRSDQVH	✓	✓	
STR67	Conophysin	CIIRNCPRGGKRDVDETHLTMPCMCCSFRQCGAPYLLWSWRMGNGDRRSDQVH		✓	
STR70	G2	DCQRGCVGCGNRAGCCCGNKYCDKDNTCQEKPAKPST	✓		
STR73	H	DSPQSECDGPRCPFICCFYEERKCGTRDCP			✓
STR76	I1	GTCSGVEQQCSNNADCCGELCCLSDKCGSPCMIRL	✓	✓	✓
STR105	M	CCIAPMCRGPCKCCEEPGHP		✓	
STR107_S3-S02	M	CCPARMCMAACSCCD		✓	
STR115_S3-G04	M	QKCCGEGSSCPKYFKNNFICGCC		✓	✓
STR116_SIIIB	M	QNCCNGGCSSKWCKGHARCC		✓	✓
STR117_SIIIA	M	QNCCNGGCSSKWCRDHARCC		✓	✓
STR144_Conotoxin-3	O1	CESYGKPCGIYNDCCNACDPAKKTCT	✓	✓	✓
STR146_SO3	O1	CKAAGKPCSRIAYNCCTGSCRSGKC		✓	✓
STR147_S6.1	O1	CKAAGKSCSRIAYNCCTGSCRSGKC		✓	✓
STR148_SVIB	O1	CKLKGQSCRKTSYDCCSGSCGRSGKC	✓	✓	
STR150	O1	CRPSGSNCGNISICCGRCVNRRCT		✓	✓
STR151_SVIA mutant 1	O1	CRPSGSPCGVTSICCGRCYRGKCT		✓	✓
STR152_SVIA	O1	CRSSGSPCGVTSICCGRCYRGKCT	✓	✓	✓
STR157	O1	DCGEGGQGCYTRPCCPGRECVAGATGGGVCL		✓	✓
STR183_S6.8	O1	DGCSNAGGFCGIHPGLCCSEICLVWCT	✓		
STR184_SVIE	O1	DGCSSGGTFCGIHPGLCCSEFCFLWCITFID			✓
STR186	O1	DKQEYHAVRKWSCVKRGDSCKTNICCAGLTCLRAHAINICLYLMPI		✓	
STR187_Mr022	O1	ECREKGQGCTNTALCCPGLECEGQSQGGLCVDN			✓
STR207	O1	KSWSCVEHGDSCKTNICCAGLTCLRAHAINLCLYLMPM		✓	✓
STR216-SO4	O1	STTKVSKATDCIEAGNYCGPTVMKICCGFCSPYSKICMNYPKN		✓	✓
STR218_SO5	O1	STTKVSKSTSCMEAGSYCGSTTRICCGYCAYFGKKCIDYPSN		✓	✓
STR221	O1	VRESDSCRKLGERCPSRPCCPRLRCGSGRAGGVCRHPYN		✓	✓
STR222	O1	VRESEGCAGLGAPCRYRRCCRRLKCVGGHVGRACRYPANYYYYY		✓	✓
STR229_contryphan-G	contryphan	GCPWEPWC		✓	✓
STR249	O2	KNCAYAFDACTSDRQCCSGYCVGNVYCE	✓	✓	✓
STR252	O2	KSNAESWWEGECRTWNAPCSFTSQCCFGKCAHHRCIAW	✓	✓	✓
STR259	O2	IYYNSARKQCLRFDYTGQGGNENNFRRTYDCQRTCLYT	✓	✓	✓
STR297	O3	DKQEYHAVRKWSCVKRGDSCKTNICCAGLTCLRARAINVCLYLMPI	✓		
STR301	O3	NTPDDGTCKSSSNCSTGQTCCKANAKNEKGFCTEDCWF		✓	
STR303	O3	TADEACKEYCEERNKNCCGRTNGEPRCASMCF		✓	✓
STR312	O3	TVDEECKEYCEQRNKNCCGETNGEPVCAQACL		✓	✓
STR320	P	RVNCAGTLCQNGKCGGDCICRPANSTHQDCQPNDFD		✓	✓
STR321	P/O2	KSNAESWWEGECRTWNAPCSSTSQCCFGRCAHHRCIAW		✓	
STR326	Sf-Mi2	DCQRGCVGCGNRAGCCCGNKYCDKDNTCQPNPVWL	✓	✓	✓
STR333	T	HCCPIDLPCCPL		✓	
STR337	W	ISKSMGDVVGRTWWCPPEGELTHAGSATKQLLSSVWGLIGGVLRMLDQNRRH	✓	✓	✓

### Comparison of venom duct transcriptomes

**Figure 1:**
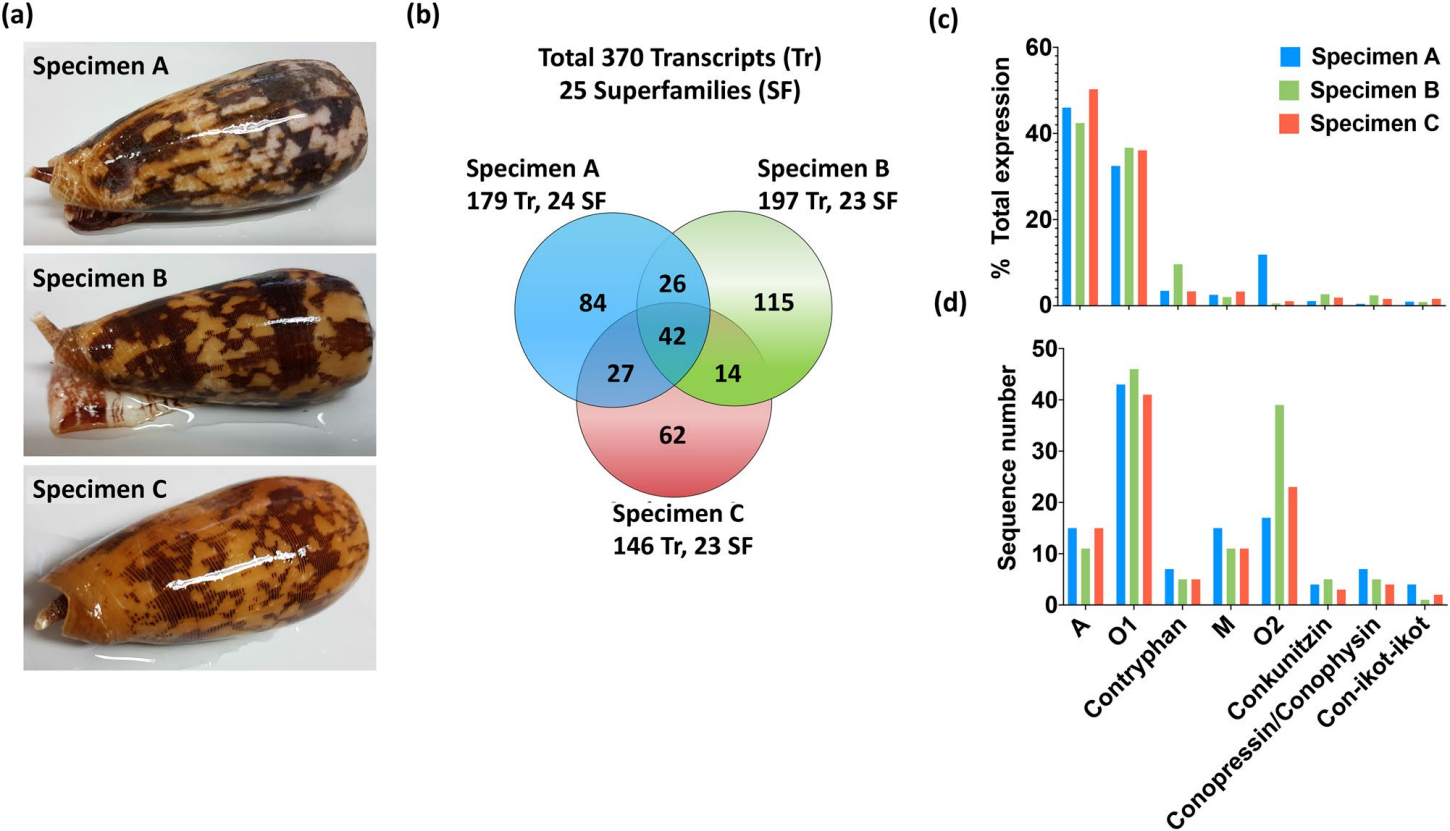
Intraspecific venom transcript variation in between three adult *C. striatus* (specimens A–C). **(a)** Photographs of the three specimens of adult *C. striatus* used in the study. **(b)** Venn diagram showing the common and unique conotoxin precursor transcripts among the three specimens of *C. striatus.* **(c)** Expression levels of the dominant conotoxin gene superfamilies across the three specimens. Relative expression levels were calculated for each superfamily using the TPM values. **(d)** The number of conotoxin precursors found in the dominant gene superfamilies.

A total of 370 unique conotoxin precursors were classified into 25 superfamilies based on the signal sequence similarity (> 60%) across the three specimens of *C. striatus* (supplementary Table S3). Despite the variety of gene superfamilies found, no new conotoxin precursor signal sequences were detected. However, high levels of variation in mature conotoxin sequences were observed, with only 11% (42) of the transcripts present in all three transcriptomes, only 29.5% (109) of transcripts shared between at least two specimens, leaving 261 unique transcripts identified form the three specimens (Fig. 1A). Only 21 of the 72 previously identified conotoxins from *C. striatus* were found in these three specimens (supplementary Table S3 and S4). This high level of intraspecific conotoxin diversity in *C. striatus* is reminiscent of the levels of variation reported in other species of cone snails from fish hunting *Pionoconus* and *Chelyconus* clades^16,24,25,28^.

Despite the variability in conotoxin precursors, the superfamily profiles of the three specimens were similar (Fig. 1B and supplementary Table S5). All three *C. striatus* transcriptomes were dominated by transcripts from superfamilies A, O1, O2, M, conkunitizin, contryphan and con-ikot-ikot, with the A and O1 superfamilies accounting for > 80% of the total conotoxin transcript expression in each specimen. In contrast, the number of transcripts from minor superfamilies was more varied (supplementary Table S5), while the O2 and M superfamilies had high sequence diversity despite their relatively low expression levels (Fig. 1C). This structural and functional conservation within superfamilies observed in *C. striatus* has been seen in other fish hunting species^16,17,26^, suggesting that diversifying selection around structurally and functionally conserved motifs underpins the evolution of conotoxin diversity in fish hunting cone snails.

### Superfamily compartmentalisation

**Figure 2:**
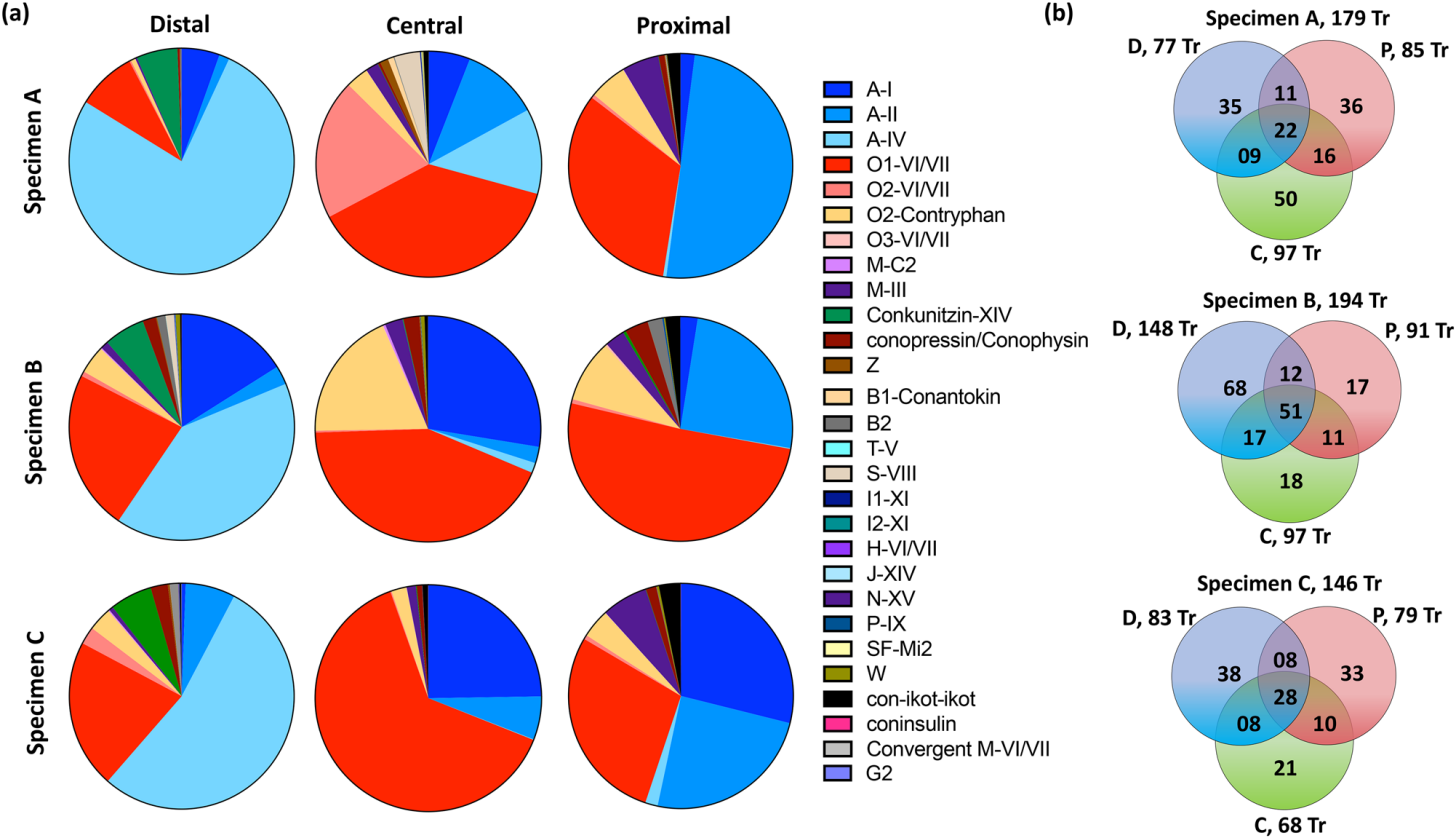
Comparison of the transcriptomic profiles of three venom duct sections (distal, central and proximal) obtained from three adult *C. striatus* (specimens A–C). **(a)** Venn diagram showing the common and unique conotoxin precursor transcripts among the three venom duct sections in three specimens of *C. striatus.* **(b)** Expression profiles (Relative TPM) of all identified superfamilies across the venom duct sections in three specimens of *C. striatus*. *Tr* transcript, *D* distal—blue, *C* central—green, *P* proximal—red.

Analysis of the conotoxin precursor transcript profiles across trisected distal, central and proximal venom duct sections revealed a clear compartmentalisation for the dominant superfamilies A, O1, O2, contryphan, conkunitzin, M and con-ikot-ikot (Fig. 2A, supplementary Table S6). Similar compartmentalisation was observed across all three specimens, suggesting that the conotoxin distribution pattern along the venom duct has functional significance. In contrast, the low and inconsistent expression levels obscured identification of any clear compartmentalisation patterns for the remaining 16 minor superfamilies. A superfamily κA-conotoxins of framework IV (A-IV) showed preferential expression in the distal venom duct (Fig. 2B), accounting for 77.3%, 43.2% and 56.2% of total relative expression distally (Fig. 2A, supplementary Figure S1) that decreased to 0.5%, 0.14% and 1.83% in the proximal section of specimens A, B and C, respectively. κA-SIVA and SIVB were the most abundant peptide precursors in all three distal transcriptomes. Similarly, conkunitzin precursors were common in the distal transcriptomes (~ 6%), while they represented < 0.1% of expression in the proximal duct sections of all three specimens. In contrast, expression levels of putative A-I and II α-conotoxins, O1, M-III and con-ikot-ikot superfamilies increased proximally in the venom duct transcriptomes of all specimens (Fig. 2A, supplementary Figure S1). Interestingly, A-I and A-II sub-families comprised ~ 50% of toxin expression in the proximal sections of specimens A and C (53% and 54.43%, respectively), but were relatively common in the distal portions in all specimens (7%, 20% and 8%, respectively). The increase in A-I and A-II toxin expression towards the proximal region is gradual, with the central section transcriptome being transitionary (Fig. 2A, supplementary Figure S1). Among A-I conotoxins, α-SI with 3/5 cysteine architecture targeting muscular nicotinic receptors was the most abundant A-I precursor (in all specimens), while α-SII precursor is the only A-II conotoxin present in all three specimens. Interestingly, α-SII was the most abundant conotoxin in the proximal transcriptomes of specimens A and B while α-SI dominates the proximal transcriptome of specimen C.

**Figure 3:**
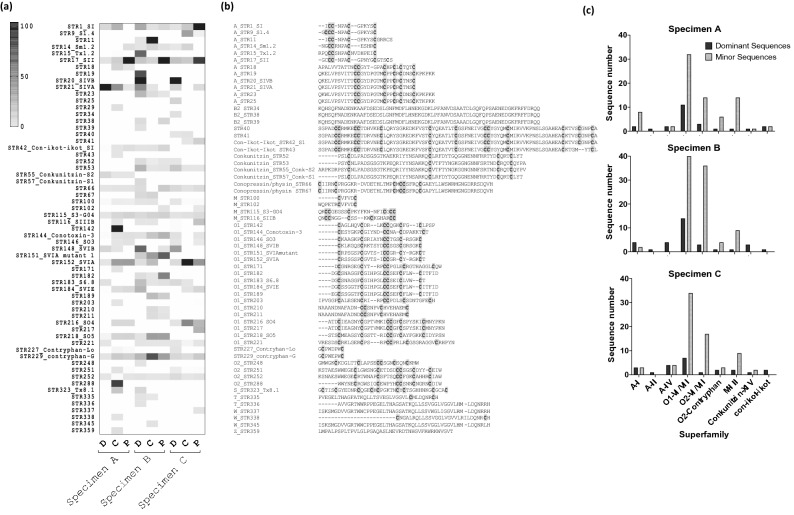
Expression level variation of the major conotoxin (relative expression level > 1%) precursors across the transcriptomes of three venom duct sections (distal, central and proximal) in three adult *C. striatus* (specimens A–C). **(a)** Heat map showing the distribution of dominant peptides across the duct section in three specimens. Relative expression levels are indicated as % TPM relative to the maximum TPM in each venom duct segment transcriptome. **(b)** The mature conotoxin sequence of the dominant sequences found across all nine transcriptomes studied in the same rank order as the heat map. **(c)** Bar graphs visualizing the contribution of the dominant and minor peptides to each main superfamily in three specimens of *C. striatus*.

The O1 superfamily transcripts were dominant in both central (40.7%, 44.7%, 64.1%) and proximal (33.3%, 54.2%, 30.1%) transcriptomes compared to the distal transcriptomes (8.5%, 24.3% 22.3%) in all three specimens. Despite these differences in the number of precursors identified, the O1 superfamily had similarly high sequence numbers and diversity across all the sections of the venom duct (supplementary Table S6). Based on the number of sequences identified, O1 was the most dominant superfamily across the venom duct, with 64 novel and 16 known ω- and δ-like conotoxins (supplementary Table S3). O1 superfamily precursor number increased proximally, except ω-conotoxin SVIB and the δ-like conotoxin S6.8 that were highly expressed in the distal transcriptomes but insignificant in the proximal transcriptomes of the three specimens (Fig. 3A). Contryphans were the third largest superfamily identified in *C. striatus* transcriptomes, with highest expression in proximal sections, among to other fish hunters studied to-date contryphans were found to be dominant in the Atlantic cone snail C*. ermineus* (*Chelyconus*)^9^, where again higher expression levels were seen proximally. The M superfamily was also highly diverse, with 24 μ-like conotoxins as well as four cysteine poor precursors related to the unclassified two-cysteine Ec2C01 from *C. emaciatus.* The expression levels of μ-like conotoxins also increased proximally (Fig. 2A) while the expression levels of cysteine poor peptides remained low along the venom duct. Among the 24 M superfamily conotoxin precursors with framework III, nine previously described μ-conotoxins and/or μ-like conotoxins were identified. However, SIIIB and S3-GO4 were the only dominant μ-conotoxins across all three specimens (Fig. 3, supplementary table S3).

### Highly abundant conotoxin precursors are conserved across individual *C. striatus*

When the conotoxin precursor profiles were compared across the duct sections of specimens A–C, only 12%, 25% and 19% of venom peptides were common to all three sections, with 32%, 48% and 36% shared between at least two duct segments, respectively (Fig. 2B). 45 of 58 (A), 74 of 91 (B) and 43 of 54 (C) of the shared precursor transcripts belonged to the dominant superfamilies O1, A-I, A-II, O2 and M. However, of the 370 conotoxin precursors found in specimens A–C, only 61 were major transcripts (> 1% of maximum TPM), including 48 belonging to these seven dominant superfamilies (Fig. 3A,B). Interestingly, most of these highly abundant transcripts were expressed along the full length of the venom duct (Fig. 3A). In contrast, of the unique peptides found in each specimen (Fig. 2B), only 9 of 121 (STRA), 11 of 103 (STRB) and 8 of 92 (STRC) were highly expressing major transcripts. This finding reveals that minor transcripts (< 1% of maximum TPM) are disproportionate drivers of intraspecific variability, while highly expressed peptides are more conserved albeit with variable levels of expression along the venom duct (Fig. 3A). When the dominant and the minor peptides are compared across the major superfamilies, it is evident that superfamilies A-I, O1, O2, M, and contryphan contribute most to this variability, with a high number of minor peptides (155) compared to the A-II, A-IV, conkunitzin and con-ikot-ikot superfamilies that have fewer minor peptides (seven) across all specimens (Fig. 3C).

### Proteomic variability

**Figure 4:**
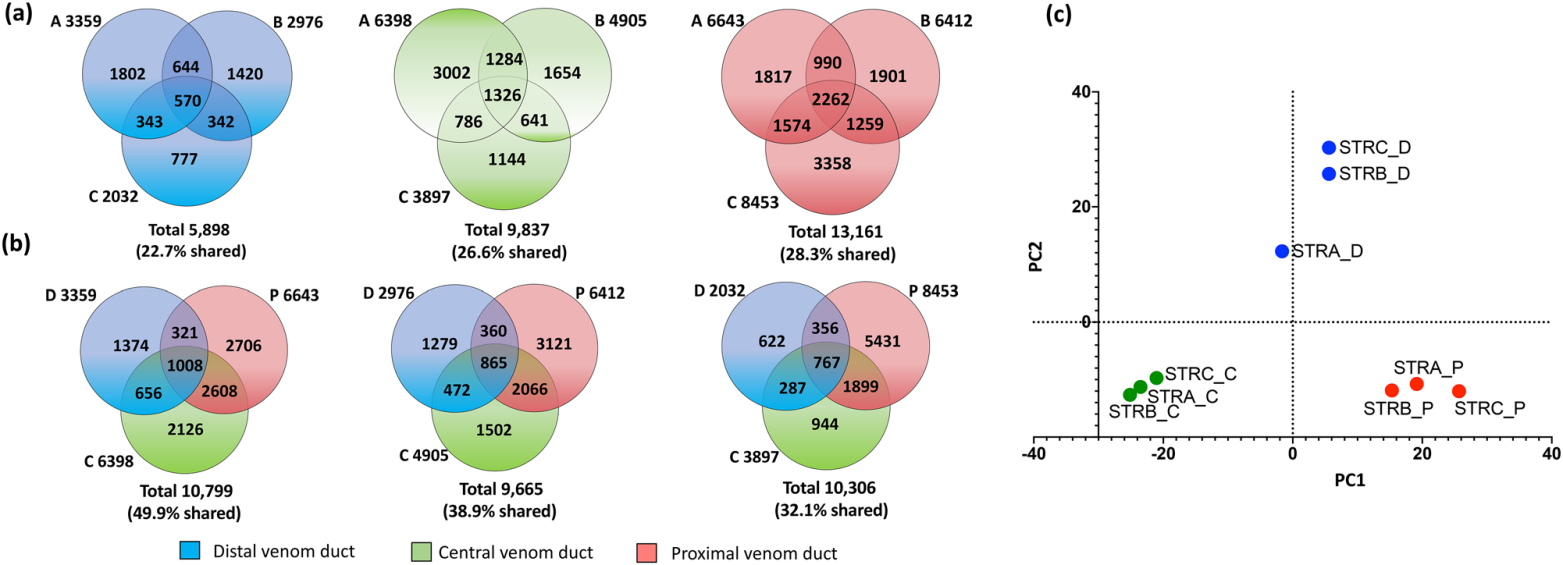
Comparison of the peptide distribution in three venom duct sections (distal, central and proximal) across the three adult *C. striatus* (specimens A–C). **(a)** Venn diagrams showing the common and unique peptide masses across the three venom duct sections (D, C and P) in three specimens (A, B and C) of *C. striatus.* Total mass units detected in the LC–MS for each specimen is shown below the respective Venn diagram. Total mass units detected in the LC–MS for each specimen is shown below the respective Venn diagram. **(b)** Venn diagrams showing the mass variability in each venom duct section (D, C and P) across the three specimens (A, B and C) of *C. striatus.* Percentage of the shared masses between the specimens are shown under the each Venn diagram. **(c)** PCA plot showing the variability of the venom extracted from dissected venom duct sections in three specimens. LC–MS data generated from SCIEX 5600 QTOF instrument is statistically analysed using the MarkerView software to obtain the PCA and loading plots.

LC–MS/MS data of the reduced alkylated and trypsin digested injected and extracted venoms from the trisected venom ducts of specimens A–C were searched against the respective conotoxin transcriptomic data to match 62 mature conotoxins belonging to 17 superfamilies in ProteinPilot (confidence value > 99%) (Table 1). Thirty three (54%) of these matched conotoxins were highly expressed in the transcriptome (Fig. 3), including 36 that were novel and currently uncharacterised. Typically proteomes of cone snails are 10–50-fold more diverse than their matching transcriptomes due to variable peptide processing and variable post translational modifications^29^. This proteomic complexity found in the relatively recently evolved cone snail venoms^16,25–27,29^ exceeds the diversity of venoms in spiders, wasps and centipedes^21^, especially when intraspecific variability is considered. Considering only the peptide monoisotopic masses ((all mentions of masses are monoisotopic unless specified otherwise) between 800–10,000 Da in extracted *C. striatus* venom duct sections the total unique masses detected were 10,799, 9665 and 10,306 in specimens A–C, respectively (Fig. 4A), consistent with the high level of diversity observed previously in cone snails^4,7,16,23,24^. Impressively, most peptide masses were unique to each specimen despite their common superfamily origins and between the three specimens a total of 18,347 unique peptide masses were detected (at a mass precision of 0.25 Da). Intraspecific venom duct peptide mass diversity was also high between comparable venom duct sections across the three specimens, with only 22.7%, 28.3% and 26.6% shared between at least two specimens in the distal, central and proximal duct sections, respectively (Fig. 4B). Proteomic data for each duct sections revealed 2-, 2.4- and 8.7-fold higher numbers of peptide masses in the proximal section of the venom duct compared to the distal in specimens A–C, respectively (Fig. 4A, supplementary Figure S2). In contrast, the central section comprised higher number of shared peptide masses with the proximal duct and fewer with distal venom duct (Fig. 4A). Overall, this profiles reflected in the conotoxin expression profiles at the transcriptomic level. (Fig. 2A).

The variability of the peptide expression patterns in the segmented venom duct LC–MS profiles (native (native undigested extracted venoms) across three specimens was statistically analysed using a supervised PCA (PC1 50% and PC2 50%). Visualised results of the PCA showed a clear differentiation between mass profiles of the three venom duct segments (Fig. 4C), while matching duct sections of each specimen clustered closely together. Localised conotoxin superfamilies and variable expression of minor peptides across and among the venom duct section may have contributed to the distances within and between these clusters. For example, distances between the distal segments were relatively high, indicative of higher levels of variability in expressed peptides in contrast to the central and proximal which clustered more tightly. Earlier findings revealed that the distal venom duct produced the predatory venom in *C. geographus* and *C. marmoreus*^4^. We propose that the high level of venom diversification seen distally in *C. striatus* may reflect its more recent origins and associated stronger diversifying selection pressures associated with the diet shift to fish hunting^4,8^.

### Venom duct origins and biochemistry of predatory and defensive venoms

**Figure 5:**
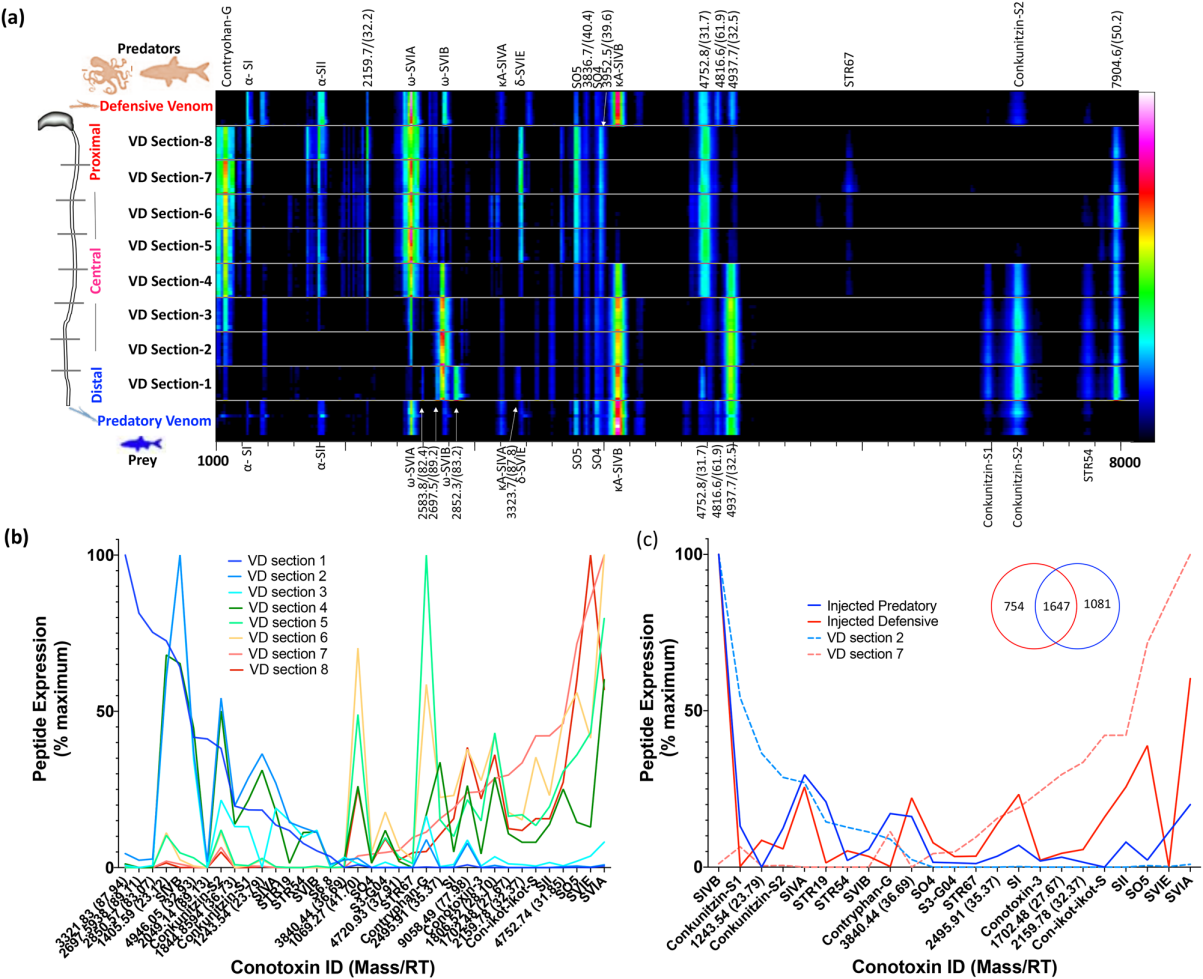
Visualisation of the venom distribution pattern across eight venom duct sections and their correlation to the injected predatory and defensive venoms obtained from the same specimen of *C. striatus* (specimen D). **(a)** MALDI spot imaging showing the alignment of the injected predatory and defensive venoms with extracted venoms of eight venom duct sections of the injected and extracted venoms. **(b)** Quantitative analysis of the major peptides found in the LC/MS profiles of the dissected venoms (venoms extracted from 8 duct segments). Both the retention time and the mass was considered when identifying the peptides across the duct sections and injected venoms. Peptides are ranked to the expression levels (relative to the maximum intensity of each sample) of section 1 (left) and 7 (right). **(c)** Mass profile comparisons were made between the LC/MS data obtained by injecting similar amount (1 μl) of the injected predatory and defensive venoms in comparison the distal duct section 2 and proximal duct section 7. Peptides are ranked by expression level (relative to the maximum intensity of each sample) of section 2 (left) and 7 (right). The inset Venn diagram shows the unique and the shared masses in each venom. Reconstructed LC/MS chromatograms were used to analyse the mass profiles and their expression levels. Figure 5b,c are also shown in heat map versions in supplementary Figure S6.

Ancestral worm-hunting cone snails are hypothesized to have repurposed defensive venoms used against predatory fish and other molluscs to facilitate prey diversification to mollusc- and fish-hunting^4,8^. The defensive venoms of worm hunters studied so far is originated in the proximal part of the venom duct^5,6^. However, the venom duct origins of the predatory and defensive venoms of hook and line fish hunting cone snails belonging to the *Pionoconus* clade has not been defined. Although collecting predatory venom from *C. striatus* is straightforward using fish as a stimulant, obtaining defensive venom has not previously been achieved. After repeated attempts on 9 specimens, we successfully collected defensive venom from *C. striatus* specimen D (supplementary Figure S3). MALDI spot imaging analysis was used to visualise the correlation of expressed peptide masses in injected predatory and defensive venoms to the extracted venoms from 8 venom duct segments from the same specimen (Fig. 5A). This analysis revealed that peptide expression profile of distal (Sections 1, 2 and 3) and proximal (Sections 6, 7 and 8) sections were distinct, while the central segments (Sections 4 and 5) shared peptide masses from both ends of the venom duct (Fig. 5A) supporting the proteomic and transcriptomic results of distal, central and proximal segments of specimens A, B and C reported above (Figs. 3A and 4A). The injected venoms and the venom duct extracts from specimen D were further analysed using Triple TOF LC/MS/MS. The expression levels of 40 most abundant peptide masses expressed across the samples were chosen to define the venom duct origins of predatory and defensive venoms. Eighteen of these 40 abundant masses were matched to highly expressed precursors in the transcriptome (Figs. 3A and 5B). However, despite their abundance, confident comparative sequence annotation for the remaining 22 abundant masses was not possible, likely due to high levels of post translational modification obscuring their transcriptomic origins.

MALDI mass imaging data and the Triple-TOF LC/MS/MS data revealed clear links between venom duct localisation and function, with most defensive venom peptides arising proximally and most predatory venom peptides arising distally, reminiscent of the pattern found in *C. geographus* and *C. marmoreous*^4^. The major venom components of the proximal venom duct included ω-conotoxins SVIA, conotoxin-3, SO4 and SO5, δ-conotoxin SVIE, α-conotoxins SI and SII, μ-conotoxin S3-GO4, con-ikot-ikot S, and conopressin/conophysin STR67 (Fig. 5B). Consistent with previous observations, these conotoxins were also highly expressed in the defensive venom (Fig. 5C) and were only present in the predatory venom at lower levels or were not detected (Fig. 5A,C). On the other hand, conkunitzin S1, conkunitzin S2 and STR54, and ω-conotoxin SVIB were more prominent in the predatory venom (Fig. 5C) and readily identified in distal duct segments (Fig. 5B). Interestingly, the distally expressed excitatory κA-SIVB, κA-SIVA, 4848.05 (30.41), 4973.09 (30.81), 4946.05 (32.93), 4752.74 (31.85) and 3840.44 (36.69) were the most abundant κA-conotoxins/κA-like glycosylated peptides in both the predatory and defensive venoms (Fig. 5C). This suggest a differential regulation of peptide expression along the venom duct can occur at the molecular level to explain its ability to be deployed in both modes of envenomation as key components. Mature κA-conotoxins are O-linked glycosylated at positions 7 (serine/tyrosine) and/or 9 (tyrosine)^30^, therefore, glycan groups (NexNAc + and Hex-HexNAc +) were screened in the injected venom proteomes to identify probable κA related peptides (Supplementary figure S4 and S5). SIVB, 4848.05 (30.41), 4973.09 (30.81), 4946.05 (32.93), 4752.74 (31.85) and 3840.44 (36.69) and SIVA were the most abundant κA-conotoxins in both the predatory and defensive venoms (Fig. 5C) despite only being expressed in the distal venom duct sections (Fig. 5A,C).

### κA-conotoxins as evolutionary driver for the successful radiation of the *Pionoconus* group?

The biochemical innovation of achieving an immediate immobility of the agile prey is suggested to be one of the driving forces of origin and success piscivory^10,16^. Excitatory δ-conotoxins found in fish hunting cone snail venoms that initiate rapid onset tetanic paralysis of the prey^16^ are believed to have underpinned the successful dietary switch to piscivory^31,32^ via repurposing the defensive venom of ancestral worm hunting species^33^. Despite the presence of δ-conotoxins, κA-conotoxins are the major class of excitatory conotoxins identified to date in the predatory venom of *Pionoconus* cone snails *C. catus*^16^, *C. consors*^34,35^ and *C. striatus*^35^, *C. striolatus*^36^ and *C. magus*^17^ reflecting the importance of their excitatory mode of action. Indeed, injection of a *C. catus* predatory venom derived mixture of κA-conotoxins (EC_50_ of 34.74 ng/g) was found to be effective in initiating tetanic paralysis in zebra fish^16^.

Given the important role of κA-conotoxins in the venoms of *Pinoconus* clade fish hunters, we explored their diversity across the fish hunting cone snail lineages. Previous phylogenetic evidence suggests that multiple independent diversification events define the evolution of mollusc hunting (*Calibanus, Cylinder, Conus, Dariconus, Eugeniconus and Leptoconus*) and fish hunting (*Phasmoconus, Gastridium, Pionoconus, Textilia, Afonsoconus, Embrikena, Asprella* and *Chelyconus*) lineages from ancestral worm hunters^12,13,37^. This evolutionary diversification was underpinned by shifts in venom chemistry, radula morphology and prey capture behaviours facilitating these significant dietary shifts. Among these new lineages, the hook and line fish hunters are the most successful in-terms of species number and distribution^12^, with the *Pionoconus* clade being the most successful with 40 known fish hunting species (supplementary Table S7).

When the available venom compositions of the representative species of the fish hunting clades are compared, κA-conotoxins are more conserved in the *Pionoconus* clade (Table 2). Three main groups have been identified based on the mature peptide sequence; full length κA-conotoxins with a conserved serine at the position 7, full length κA-conotoxins with a conserved tyrosine at the positions 7 and 9, and relatively short κA-conotoxins. Bu27 from the *C. bullatus* (*Textila*) transcriptome is the only full length κA-conotoxin (tyrosine at the positions 7 and 9) identified outside the *Pionoconus* clade^38^. Interestingly the *Pionoconus* and *Textila* are sister clades^12^, while the short excitatory κA-conotoxins have only been reported in Atlantic fish hunting *C. ermineus*^9^ and *C. purpuracens*^39^ of the distinct *Chelyconus* clade (Table 2). Long κA-conotoxins are also not found in net hunters (*C. geographus* and *C. tulipa*) and other linages of suggested fish hunters (*Phasmoconus, Afonsoconus, Embrikena* and *Asprella*), or mollusc hunting or worm hunting clades investigated to-date, suggesting they have evolved relatively recently.

**Table 2:**
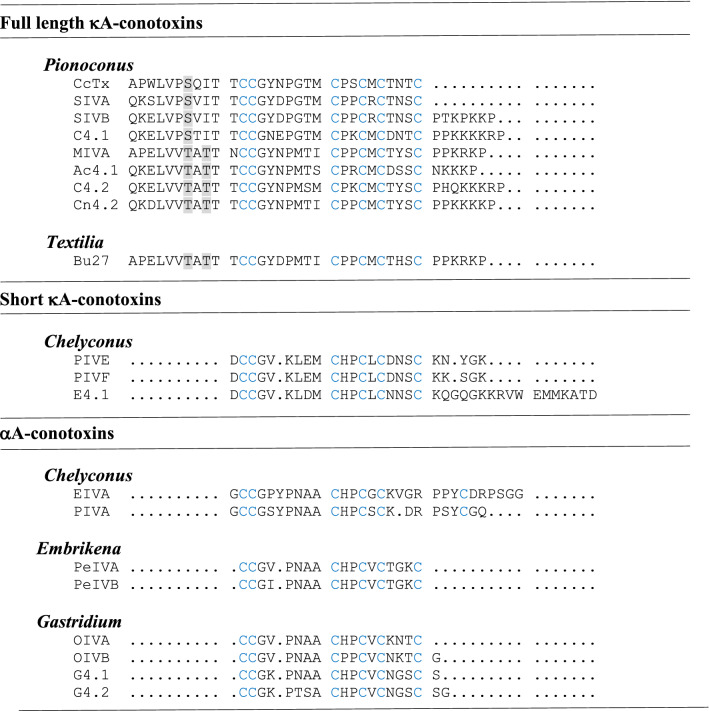
Complexity and the distribution of the A superfamily Cysteine framework IV peptides across fish hunting clades of cone snails.

Most A superfamily peptides have two disulphide bonds and target nicotinic receptors^2,40^. Through gene duplication and positive selection, the A superfamily successfully incorporated a third disulphide bond seen in framework IV αA-conotoxins and κA-conotoxins (Table 2). αA-conotoxins that target muscular nAChRs have been found in *C. ermineus* (*Chelyconus*)^9^*, C. purpuracens* (*Chelyconus*)^41^*, C. pergrandis* (*Embrikena*)^42^*, C. geographus* (*Gastridium*)^42^ and *C. obscurus* (*Gastridium*)^43^. However, no αA-conotoxins have been identified in cone snails of the *Pionoconus* clade where κA-conotoxins dominate. This clear separation in the presence of αA and κA conotoxins in different lineages of fish hunters (Table 2) likely reflects distinct evolutionary pressures associated with the dietary shift to fish hunting in cone snails with different hunting strategies^44^. Short excitatory κA conotoxins found in fish hunting *Chelyconus* species with structural similarity to αA-conotoxins may present an example of early functional divergence from paralytic to excitatory peptides.

## Conclusions

This study reports the first characterisation of venom composition across the venom duct of *C. striatus* and its correlation to the respective injected predatory and defensive venoms. A clear venom duct compartmentalisation was observed at both the transcriptomic and proteomic level with peptides belonging to neuromuscular inhibitors of A, O1 and M are highly expressed in the proximal region compared to the distal region and excitatory peptides (κA and conkunitzins) are distally dominant. This study revealed that these major superfamily conotoxins dominated the *C. striatus* venom duct are thus likely to be critical for survival and appear to be subjected to purifying selection. In contrast, minor superfamilies were disproportionately more variable and thus less likely to be critical for survival and appear to be subjected to diversifying selection.

Having κA-conotoxins exclusively produced in the distal segment suggest that during adaptation to fish hunting, toxin classes optimised for fish hunting have supplanted predatory worm hunting toxins in the distal venom duct section, perhaps through repurposing of defensive venoms through adaptive predator–prey evolutionary mechanisms as previously explained for other venomous animals^45,46^. This hypothesis can also be adapted to net feeding fish hunters *C. geographus* and *C. tulipa* where the distal venom duct sections are enriched with components used for net feeding^4,26^. In contrast, *Pionoconus* clade cone snails uniquely express high level of κA-conotoxins to support their hook and line hunting behaviour. Based on their expression pattern and mode of action, we propose that κA-conotoxins are the key evolutionary innovation underpinning the explosive adaptive radiation seen for *Pionoconus* clade fish hunters. Our findings highlight the need to study additional clades of cone snails to determine how the spatial distribution of conotoxins along the venom duct correlate with injected predatory and defensive venoms. Further studies on understanding the molecular mechanisms, gene structures and hypermutation events contributing to divergence events are required to better understand molecular evolution trajectories in these venomous animals.

## Methods

### Venom collection

Four adult specimens of *C. striatus* collected (at the same time) from Coral Reef in the Northern Great Barrier Reef, Australia were used for the study. Snails were housed in marine aquarium at 24–28 °C and a 12:12 light–dark cycle. Milked predatory venoms were collected from all specimens using cadaver zebrafish as a stimulant and lyophilized and stored at –20 °C until use. Three specimens (A, B and C) (Fig. 1D) were sacrificed and the distal (D), central (C) and proximal (P) thirds stripped of cellular content and apportioned for RNA extraction (75%) and proteomic (25%) studies. Injected defensive venom from specimen D was collected by inducing defensive behaviour using a combination of chemical (using a predatory cone snail *C. textile*) and physical (pushing the foot using a blunt forceps) stimuli, as previously described^4^, whereas the other specimens were unresponsive. Specimen D was scarified 1 week after the last milking and the venom duct dissected into 8 equal portions and the cellular contents stripped for proteomic analysis.

### Venom extraction from dissected venom ducts

In house optimised method for conotoxin extraction from dissected cone snail venom ducts was used. Stripped venom duct cells from each section were triturated with 500 μl chilled 30% acetonitrile (ACN) containing 1% formic acid (FA) and centrifuged for 20 min at 12,000 × *g*. The supernatant was removed and spun at 12,000 × *g* for another 10 min to separate the fine insoluble material from the crude venom. Protein estimates were obtained using NanoDrop (Thermo Fisher Scientific) A280 method. The venom extracts were immediately lyophilised until further use.

### RNA extraction and transcriptomic sequencing

To extract total RNA, venom duct cells collected as described above were placed in a 1.5 mL tube containing 0.5 mL of TRIzol reagent (Invitrogen) and total RNA extracted following the manufacturer’s instructions to yield 5–10 μg of purified total RNA from each section (Table 1). Sequencing libraries were prepared from total RNA using the TruSeq Stranded mRNA Sample Prep Kit with TruSeq library indices (Illumina Inc., San Diego, CA, USA) following the manufacturer’s instructions. The sequencing libraries obtained were pooled and sequenced on a 2 × 150 bp High Output Kit v2 run on a NextSeq 500 machine (Illumina Inc., San Diego, CA, USA). The acquired transcriptomic data was processed using *BBMap* tools (version 38.00 from https://jgi.doe.gov/data-and-tools/bbtools/) to remove sequencing adaptors, low quality reads (Phred score < 28), and the reads of < 50 bases (bbduck.sh in1 = R1.fastq in2 = R2.fastq out1 = outputR1.fastq out2 = outputR2.fastq ref = seqadaptorslist.fa qtrim = rl trimq = 28 ftl = 10 minlen = 50). The filtered datasets from each section were then merged as paired end reads using *BBMap tools* (bbmerge.sh in1 = outputR1.fastq in2 = outputR2.fastq out = merged.fastq outu1 = unmerged_outputR1.fastq outu2 = unmerged_outputR2.fastq) and then the merged and unmerged reads were assembled as a single reads using *Trinity-v2.8.4* (https://github.com/trinityrnaseq/trinityrnase) using a kmer size of –19 and –31, a maximum chrysalis cluster size of 40, with no Butterfly transcript reduction parameters set to better identify low level transcripts and transcript variants as described before^36^. The assembled transcripts from both kmers were merged in a single dataset and the duplicate transcripts were removed using *BBMap* tools (dedupe.sh in = merged_assembled_transcripts.fasta out = duplicates_removed_transcripts.fasta). The candidate conotoxins and conopeptides were identified as previously described^47^ using the latest available versions of the software. After ConoSorter analysis, sequences with signal peptides were retrieved by the signalP4.1 server (http://www.cbs.dtu.dk/services/SignalP-4.1/) using default parameters. The retrieved sequences were submitted to BLASTp (*blast* + version 2.4.0, e-value = 0.75) against the non-redundant *UniProt* database before classifying the sequences into superfamily. To classify the sequences into the superfamilies, the sequences were classified based on their signal sequences using *cd-hit-v4.6.7* (https://github.com/weizhongli/cdhit/releases/tag/V4.6.7) with signal sequence identity > 75%. Finally, the annotated conotoxin transcripts were quantified using *salmon v0.11.3* (https://github.com/COMBINE-lab/salmon) with default parameter settings. All the computational analyses were performed in high performance computing cluster in PBS Pro environment.

ConoPrec software (http://www.conoserver.org) was then used to identify the conserved signal sequences, cysteine frameworks, cleavage sites and previously reported conotoxins^48^. During this process, precursors < 40 amino acids in length, signal sequence hydrophobicity less than 40%, and repeated sequences were manually removed. Considering the published variations in signal conservation within superfamilies, the cut-off value used to assign a gene superfamily was set as 53.3%^49^.

### Reduction alkylation and trypsin digestion

Aliquots of collected venom (50 μg) were lyophilized and reconstituted in 50 μL of freshly prepared 100 mM NH_4_HCO_3_ in 30% acetonitrile at pH 8 prior to reduction and alkylation using the previously described triethylphosphine/iodoethanol protocol^50^. Sigma proteomics sequencing grade trypsin was used for enzyme digestion of reduced and alkylated peptides as described^27,29^.

### Mass spectrometry (MS)

#### LC–ESI–MS/MS

Native injected and dissected venoms, reduced alkylated venoms and trypsin digested venoms were centrifuged (12,000 × *g*) to remove particulate matter prior to liquid chromatography-electrospray mass spectrometry (LC–ESI–MS) performed on an Sciex TripleTOF 5600 instrument coupled to a Shimadzu 30 series HPLC. HPLC separation was achieved on a Zorbax C18 4.6 × 150 mm column using a linear 1.3% B (acetonitrile/0.1% formic acid (aq) min^–1^ gradient at a flow rate of 0.2 ml min^–1^ over 90 min. The gradient is optimised to elute the hydrophilic components in the beginning and hydrophobic protein like components towards the end of the run to capture the toxin peptides eluting in between. A cycle of one full scan of the mass range (MS) (300–2000 *m/z*) followed by multiple tandem mass spectra (MS/MS) was applied using a rolling collision energy relative to the *m/z* and charge state of the precursor ion up to a maximum of 80 eV.

### ConoServer and ProteinPilot search of LC/MS and LC/MS/MS data

LC–ESI–MS reconstruction of the native venom samples were performed using Analyst LCMS reconstruct BioTools (Framingham, MA, USA) with the mass range set to 800–10,000 Da, a mass tolerance of 0.2 Da, and S/N threshold set to 10. The LC/MS reconstruct compute the monoisotopic masses and the list of monoisotopic masses between 800 and 10,000 Da were compared between the venom duct sections and specimens. The monoisotopic mass lists were submitted to “remove duplicates” tool of ConoServer to remove duplicated masses (0.5 Da mass tolerance)^48^. To increases the chances of detecting post translationally modified conotoxins and to complement the ProteinPilot search “differential PTM mass” tool of ConoServer was used to calculate the monoisotopic masses of the identified mature conotoxins of the transcriptome with predicted PTMs. Then these calculated monoisotopic masses with predicted PTMs were matched to the duplicate removed monoisotopic mass list obtained from the LC–MS reconstruct. The precision level was set to 0.25 Da for automatic matching search.

The ProteinPilot 5.0 software (SCIEX, Framingham, MA, USA) was used to search the LC–ESI–MS/MS mass lists (mass tolerance of 0.05 Da) to identify precursor ions in reduced/alkylated and reduced/alkylated and reduced/alkylated-trypsin digested venom extracts from three venom duct sections of all three specimens. These mass data bases from each segment were separately matched against the *C. striatus* venom duct transcriptome sequences (370) obtained for all venom duct sections. The ProteinPilot Search was done separately using the reduced/alkylated and reduced/alkylated-trypsin digested venom extracts. Posttranslational modifications (PTM) used in the search covered amidation, deamidation, hydroxylation of proline and valine, oxidation of methionine, carboxylation of glutamic acid, cyclization of N-terminal glutamine (pyroglutamate), bromination of tryptophan, sulfation of tyrosine. Additionally O-glycosylation PTMs was included in the search as typical sugar ions associated with glycosylation was identified in the MS spectrum (supplementary Figure S4). The threshold confidence value for accepting identified spectra was set to 99 and identified fragment masses were searched manually to confirm assignment. Given the challenges sequencing venom peptides with PTMs from proteomic data, we have relied on venom transcriptomic analysis to determine the distribution of venom peptides across the venom duct, with support from complementary proteomic identification of major toxins and toxin-related masses.

### MALDI spot imaging

The extracts of the duct sections were analysed using an Ultraflex III TOFTOF (time-of-flight) mass spectrometer (Bruker Daltonics, Bremen, Germany) equipped with a 200 Hz all-solid-state laser (SmartBeam) and controlled by the FlexControl 2.4 software package using a previously reported method for cone snail venom analysis^4^. To analyse peptides with a mass range of 1000 Da and 10,000 Da, Ultraflex III was operated in both linear-positive and reflectron-positive mode using CHCA as a matrix. Spectra calibration was performed externally using a peptide calibration mixture (206195, Bruker Daltonics, Bremen, Germany). A CHCA solution was made by the dilution of acetone saturated with CHCA 1 in 10 with an acetone:acetonitrile:water (6:3:1) solution. The raw samples were diluted 1 in 100 with 0.1% TFA, and 2 ml of diluted matrix solution mixed with 1 ml sample and spotted onto a polished steel target. For all samples, 400 shots were acquired using a random walk function at a laser frequency of 200 Hz and saved, with 10 replicates of each sample averaged^4^. Data were loaded into Clinprot Tools (Bruker Daltonics, Bremen, Germany) to visualize the 8 individual duct sections and injected predatory and defensive venoms in ‘gel view’ using a colorimetric gradient to show the abundance of the components in respective fractions.

### Mass spectrometric data visualisation

Reconstructed mass lists from LC–ESI–MS runs of the native injected and dissected venom samples were further processed to remove Na^+^ and K^+^ adducts and remove duplicate masses using the embedded tools in ConoServer^45^. The processed LC/MS mass lists containing the monoisotopic mass, retention time and relative intensity were imported into the MarkerView (version 1.3.1) software (Sciex, Framingham, MA) to generate the proteome matrix comprising a list highly expressing peptide masses present at least in two samples across the venom duct extracts (distal, central and proxomal) of three specimens (A, B and C). Relative intensities (percentage of maximum) were generated as a percentage of the most abundant peptide in each individual venom using Analyst (version 1.6) software, with unique masses aligned according to the retention time and filtered to remove background ions. Data alignment algorithms in MarkerView software were then applied to compensate for minor variations in mass and retention time to ensure the same compounds were accurately identified across the samples using a noise of threshold 10, minimum spectral peak width of 5 ppm, maximum RT peak width at 100 scans, a retention time tolerance of 0.5 min, a mass tolerance of 25 ppm, and a maximum number of peaks to 1000^25^. The generated peak list and their relative abundances was used as the data matrix (supplementary Table S9) for principal component analysis (PCA) to visualize the clustering patterns of venom duct sections in replicated specimens in a Scores plot.

## Supplementary Information

Supplementary Information.

Supplementary Table S3.

Supplementary Table S8.

Supplementary Table S9.

Supplementary Dataset.

## Data Availability

Data files of the reduced alkylated and reduced alkylated-trypsin digested samples are deposited in the Proteomics Identification Database (PRIDE) of EMBL-EBI (accession number PXD026194). Raw data files of the specimens A venom duct transcriptomic sequences are deposited in Sequence Read Archive (SRA) of NCBI (accession number PRJNA730990).
