# Supplementary material for: Venom duct origins of prey capture and defensive conotoxins in piscivorous Conus striatus
Source: Sci Rep. 2021 Jun 24;11:13282. doi: 10.1038/s41598-021-91919-4 (PMC8225645; doi:10.1038/s41598-021-91919-4)
Supplement: Supplementary file 1 — Supplementary Information. [file 41598_2021_91919_MOESM1_ESM.docx]

**Supplementary Information**

**Venom duct origins of prey capture and defensive conotoxins in piscivorous *Conus striatus***

Himaya S.W.A.^a^, Ai-Hua Jin^a#^, Brett Hamilton^b#^, Subash K. Rai ^a,c#^, Paul Alewood ^a^, Richard Lewis ^a*^

^a^ Institute for Molecular Bioscience, The University of Queensland, St Lucia, 4072 Queensland, Australia

^b^ Centre for Microscopy and Microanalysis, The University of Queensland, St Lucia, QLD 4072, Australia

^c^ Current address: UQ Genome Innovation Hub, Institute for Molecular Bioscience, The University of Queensland, St Lucia, 4072 Queensland, Australia

^#^These authors contributed equally to this study

^*^ Corresponding author

**Supplementary Table S1. Summary statistics of the transcriptome analysis outcome**

| **Transcriptome ID** | **RNA Yield (μg)** | | **Raw reads**  **(After cleaning and trimming** | **Number of predicted conotoxin transcripts** | **Number**  **of annotated conotoxin precursors** | **Number of**  **conotoxin superfamilies** |  |
| --- | --- | --- | --- | --- | --- | --- | --- |
| Specimen A_Distal | | 10.31 | 54842354 | 1777 | 77 | 19 |  |
| Specimen A_Central | | 7.93 | 38640008 | 1916 | 97 | 22 |  |
| Specimen A_Proximal | | 12.56 | 31850880 | 1100 | 85 | 19 |  |
| Specimen B_Distal | | 8.76 | 24804145 | 1765 | 148 | 23 |  |
| Specimen B_Central | | 5.78 | 23542981 | 1609 | 97 | 20 |  |
| Specimen B_Proximal | | 4.92 | 30241824 | 1499 | 91 | 20 |  |
| Specimen C_Distal | | 4.61 | 25689808 | 1707 | 83 | 20 |  |
| Specimen C_Central | | 5.68 | 29064106 | 1401 | 68 | 19 |  |
| Specimen C_Proximal | | 5.26 | 25689808 | 1324 | 79 | 21 |  |
|  | |  |  |  |  |  |  |

**Supplementary Table S2:** Superfamily distribution and precursor number of each superfamily found from the assembled data using default trinity parameters.

|  | **Specimen A** | | | **Specimen B** | | | **Specimen C** | | |
| --- | --- | --- | --- | --- | --- | --- | --- | --- | --- |
| **Superfamily** | **Distal** | **Central** | **Proximal** | **Distal** | **Central** | **Proximal** | **Distal** | **Central** | **Proximal** |
| **A** | 10 | 13 | 16 | 12 | 10 | 11 | 8 | 11 | 7 |
| **O1** | 18 | 13 | 22 | 14 | 16 | 16 | 15 | 13 | 10 |
| **M** | 7 | 10 | 13 | 14 | 18 | 16 | 10 | 5 | 10 |
| **O2** | 4 | 7 | 8 | 23 | 11 | 16 | 5 | 7 | 13 |
| **T** | 5 | 9 | 12 | 15 | 10 | 5 | 6 | 1 | 3 |
| **O3** | 1 | 2 | 3 | 2 | 1 | 2 | 3 | 1 | 1 |
| **O4** | 0 | 0 | 0 | 2 | 0 | 0 | 0 | 0 | 0 |
| **H** | 2 | 3 | 3 | 3 | 2 | 3 | 3 | 3 | 3 |
| **I1** | 2 | 3 | 3 | 6 | 1 | 1 | 2 | 2 | 2 |
| **B1** | 1 | 0 | 0 | 2 | 3 | 3 | 3 | 2 | 2 |
| **I2** | 1 | 0 | 0 | 1 | 2 | 3 | 2 | 0 | 0 |
| **P** | 1 | 2 | 2 | 0 | 0 | 0 | 0 | 0 | 0 |
| **R** | 1 | 1 | 1 | 1 | 1 | 1 | 1 | 1 | 0 |
| **S** | 2 | 5 | 6 | 3 | 3 | 1 | 1 | 1 | 1 |
| **SF-mi2** | 1 | 0 | 0 | 0 | 0 | 0 | 0 | 0 | 1 |
| **SF-mi5** | 1 | 1 | 1 | 1 | 1 | 1 | 1 | 1 | 1 |

**Supplementary Table S3. (Attached as a separate spreadsheet)** Full report of the venom transcripts identified from three specimens of *C. striatus* and their expression levels.

**Supplementary Table S4.** Summary of the known vs novel conotoxins identified from the *C. striatus* trascriptome. (Known conotoxins identified in the current transcriptome are shown in bold letters in column 2)

|  | **Previously identified Conotoxins** | | **Conotoxins identified in the current study** | |
| --- | --- | --- | --- | --- |
| **Superfamily** | **Sequence ID** | **Sequence #** | **Sequence ID** | **Total Sequence #** |
| A | **SI**, SIA, **SII**, **SIVA**, **SIVB**, S1.1, S1.4, S1.6, S1.7, S1.8, S1.9, S1.10, S4.3, S4.4 | 14 | STR1_SI, STR2, STR3, STR4, STR5, STR6_Ec1.8, STR7, STR8_TxIA, STR9_S1.4, STR10, STR11, STR12, STR13, STR14_Sm1.2, STR15_Tx1.2, STR16_Ec1.6, STR17_SII, STR18, STR19, STR20_SIVB, STR21_SIVA, STR22, STR23, STR24_Sx4.1, STR25, STR26, STR27,STR28 | 28 |
| B1 |  | NA | STR29, STR30, STR31, STR32, STR33 | 5 |
| B2 |  | NA | STR34, STR35, STR36, STR37, STR38, STR39 | 6 |
| Contryphan | Contryphan-S | 1 | STR226, STR227_Contryphan-Lo, STR228, STR229_contryphan-G, STR230, STR231, STR232, STR233, STR234, STR235, STR236 | 11 |
| Con-ikot-ikot | **Con-ikot-ikot_S1** | 1 | STR40, STR41, STR42_Con-ikot-ikot_S1, STR43, STR44, STR45 | 6 |
| Coninsulin |  | NA | STR46, STR47, STR48, STR49, STR50, STR51 | 6 |
| Conkunitzin | **Conkunitzin-S1**, **Conkunitzin-S2** | 2 | STR52, STR53, STR54, STR55_Conkunitzin-S2, STR56_Conkunitzin-S1, STR57, STR58, STR59 | 8 |
| Conopressin/ Conophysin | **Conopressin-S** | 1 | STR60, STR61, STR62, STR63, STR64, STR65, STR66, STR67, STR68, STR69 | 10 |
| G2 |  | NA | STR70, STR71, STR72 | 3 |
| H |  | NA | STR73, STR74, STR75 | 3 |
| I1 | S11.1, S11.2a | 2 | STR76, STR77, STR78, STR79_Tx11.3, STR80, STR81, STR82, STR83_S11.1, STR84, STR85, STR86 | 11 |
| I2 | S11.3 | 1 | STR87, STR88, STR89, STR90, STR91, STR92_TxXI, STR93, STR94 | 8 |
| J |  | NA | STR95, STR96, STR97, STR98 | 4 |
| M | **SIIIA**, **SIIIB**, S14-H01, S3-D01, S3-E02, S3-E03, **S3-G04**, S3-I01, S3-I05, S3-KP02, S3-L02, S3-S01, **S3-S02**, S3-TS01, S3-VP01, S3-WP01, S3-Y01 | 17 | STR99, STR100, STR101_Ec2C01, STR102, STR103, STR104_Rt3-WP01, STR105, STR106, STR107_S3-S02, STR108, STR109, STR110_Mi3-T02, STR111, STR112, STR113_Rt3-E05, STR114, STR115_S3-G04, STR116_SIIIB, STR117_SIIIA, STR118, STR119, STR120, STR121, STR122, STR123, STR124, STR125_Vr3-Q01 | 27 |
| M---L-LTVA |  | NA | STR126, STR127, STR128_Vc7.3, STR129, STR130, STR131, STR132 | 7 |
| N |  | NA | STR133, STR134, STR135, STR136, STR137, STR138, STR139, STR140, STR141 | 9 |
| O1 | **SVIA**, **SVIA mutant 1**, **SVIB**, **SVIE**, **SO3**, **SO4**, **SO5**, Conotoxin-15, Conotoxin-2, **Conotoxin-3**, Conotoxin-9, S6.1, S6.10, S6.2, S6.6, S6.7, **S6.8** | 17 | STR142, STR143_King-Kong 2, STR144_Conotoxin-3, STR145_TxMKLT1-015, STR146_SO3, STR147_S6.1, STR148_SVIB, STR149_TxO1, STR150, STR151_SVIA mutant 1, STR152_SVIA, STR153, STR154, STR155, STR156_TxO2, STR157, STR158, STR159, STR160, STR161, STR162, STR163, STR164, STR165, STR166, STR167, STR168, STR169, STR170, STR171, STR172, STR173, STR174, STR175, STR176, STR177, STR178, STR179, STR180, STR181, STR182, STR183_S6.8, STR184_SVIE, STR185, STR186, STR187_Mr022, STR188, STR189, STR190, STR191, STR192, STR193, STR194, STR195, STR196, STR197, STR198, STR199, STR200, STR201, STR202, STR203, STR204, STR205, STR206, STR207, STR208, STR209, STR210, STR211, STR212, STR213, STR214, STR215, STR216-SO4, STR217, STR218_SO5, STR219,STR220, STR221, STR222, STR223, STR224_TxVIA, STR225 | 84 |
| O2 | S15a, S6.11 | 2 | STR237, STR238, STR239, STR240_TxMEKL-P2, STR241, STR242, STR243, STR244, STR245, STR246, STR247, STR248, STR249, STR250, STR251, STR252, STR253, STR254, STR255, STR256, STR257, STR258, STR259, STR260, STR261, STR262, STR263, STR264, STR265, STR266, STR267, STR268, STR269, STR270, STR271, STR272, STR273, STR274, STR275, STR276, STR277, STR278, STR279, STR280, STR281, STR282, STR283, STR284, STR285, STR286, STR287, STR288, STR289 | 53 |
| O3 | S6.16, S6.17, S6.18 | 3 | STR291, STR292, STR293, STR294, STR295, STR296, STR297, STR298, STR299, STR300, STR301, STR302, STR303, STR304, STR305, STR306, STR307, STR308, STR309, STR310, STR311, STR312, STR313, STR314, STR315, STR316, STR317, STR318 | 29 |
| P |  | NA | STR319_TxIXA, STR320, STR321, STR322 | 4 |
| S | S8.1, S8.2 | 2 | STR323_Tx8.1 | 1 |
| SF-mi2 |  | NA | STR324, STR325, STR326 | 3 |
| T | S10.1, S5.1, S5.2. S5.3 | 4 | STR327_S5.2, STR328_Eb5.5, STR329_TeA31, STR330, STR331, STR332, STR333, STR334 | 8 |
| W |  | NA | STR335, STR336, STR337, STR338, STR339, STR340, STR341, STR342, STR343, STR344, STR345, STR346, STR347, STR348, STR349 | 15 |
| Z |  | NA | STR350, STR351, STR352, STR353, STR354, STR355, STR356, STR357, STR358, STR359, STR360, STR361, STR362, STR363, STR364, STR365, STR366, STR367, STR368, STR369, STR370 | 21 |

**Supplementary Table S5.** Transcript number and expression level of superfamilies across the n three specimens of *C. striatus.* Expression levels are indicated as a %TPM relative to the total expression in each specimen.

| **Specimen ID** | **Specimen A** | | **Specimen B** | | **Specimen C** | |  |
| --- | --- | --- | --- | --- | --- | --- | --- |
| **Superfamily** | Transcript Number | Expression level (% TPM) | Transcript Number | Expression level (% TPM) | Transcript Number | Expression level (% TPM) |  |
| A-I | 10 | 4.963 | 6 | 15.542 | 6 | 19.107 |  |
| A-II | 1 | 20.262 | 1 | 8.991 | 1 | 14.055 |  |
| A-IV | 4 | 20.768 | 4 | 17.878 | 8 | 17.109 |  |
| O1 | 43 | 32.463 | 46 | 36.677 | 41 | 36.095 |  |
| Contryphan | 7 | 3.480 | 5 | 9.654 | 5 | 3.338 |  |
| O2 | 17 | 11.865 | 39 | 0.545 | 18 | 1.070 |  |
| O3 | 11 | 0.023 | 14 | 0.132 | 11 | 0.033 |  |
| M-C2 | 2 | 0.025 | 2 | 0.157 | 4 | 0.070 |  |
| M-III | 15 | 2.547 | 10 | 2.018 | 11 | 3.302 |  |
| Conkunitzin | 2 | 1.100 | 5 | 2.675 | 4 | 1.905 |  |
| T | 5 | 0.002 | 6 | 0.013 | 0 | 0.000 |  |
| conopressin/conophysin | 7 | 0.470 | 5 | 2.426 | 4 | 1.580 |  |
| S | 1 | 2.148 | 1 | 0.513 | 0 | 0.000 |  |
| I1 | 5 | 0.092 | 7 | 0.041 | 3 | 0.018 |  |
| I2 | 3 | 0.005 | 2 | 0.002 | 4 | 0.002 |  |
| B1 | 4 | 0.510 | 0 | 0.000 | 1 | 0.005 |  |
| B2 | 3 | 0.094 | 3 | 1.120 | 1 | 0.369 |  |
| G2 | 0 | 0.000 | 0 | 0.000 | 4 | 0.020 |  |
| H | 1 | 0.000 | 3 | 0.006 | 1 | 0.008 |  |
| J | 3 | 0.000 | 4 | 0.022 | 1 | 0.002 |  |
| N | 2 | 0.008 | 4 | 0.013 | 6 | 0.026 |  |
| P | 2 | 0.020 | 3 | 0.113 | 1 | 0.033 |  |
| SF-Mi2 | 3 | 0.032 | 1 | 0.000 | 1 | 0.001 |  |
| W | 3 | 0.181 | 11 | 0.477 | 1 | 0.140 |  |
| Z | 11 | 0.735 | 10 | 0.116 | 4 | 0.092 |  |
| con-ikot-ikot | 4 | 0.962 | 1 | 0.863 | 2 | 1.604 |  |
| coninsulin | 3 | 0.002 | 4 | 0.003 | 1 | 0.010 |  |
| M---L-LTVA | 7 | 0.006 | 2 | 0.001 | 4 | 0.007 |  |
|  |  |  |  |  |  |  |  |

**Supplementary Table S6.** Transcript number and expression level of major superfamilies across the three venom duct segments in three specimens of *C. striatus.* Expression levels are indicated as a %TPM relative to the maximum expression in each venom duct section (not the total expression)

**Supplementary Table S7.** Species and diet distribution in the fish hunting clades of cone snails

*Direct evidence on the dietary mode has not been collected

| **Clade** | **Type Species** | **Fish hunting mode** | **Total Species** | **Sub-Species** | **Fish Hunting Species** | **Worm Hunting Species** | **Diet Un-identified** |
| --- | --- | --- | --- | --- | --- | --- | --- |
| *Pionoconus* | *C. magus* | Harpoon | 41 | 6 | 40 | 7 | - |
| *Phasmoconus* | *C. radiatus* | Harpoon | 86 | 3 | 12 | 20 | 53 |
| *Gastridium* | *C. geographus* | Harpoon/Net | 6 | - | 6 | - | - |
| *Textilia* | *C. bullatus* | Harpoon | 11 | - | 11 | - | - |
| *Chelyconus* | *C. ermineus* | Harpoon | 2 | 1 | 3 | - | - |
| *Embrikena** | *C. pergrandis* | Not reported | 3 | - | ? | 3 | - |
| *Asprella** | *C. sulcatus* | Not reported | 9 | - | ? | 8 | 1 |
| *Afonsoconus** | *C. kinoshitai* | Not reported | 4 | - | ? | 4 | - |

**Supplementary Figure S1.** Expression pattern (Relative TPM compared to maximum conotoxin TPM of each transcriptome) of dominant superfamilies across the venom duct sections in three specimens of *C. striatus*.

**Supplementary Figure S2.** LC/MS chromatogram of extracted native venom from each duct section of *C. striatus* specimen A.

******

**Supplementary Figure S3.** TIC chromatograms of the injected predatory and defensive venom collected from *C. striatus* specimen D.

**Supplementary Figure S4.** Evidences of the presence of carbohydrate marker ions in the TIC of C*. striatus* injected venoms. The extracted chromatograms (XIC) corresponding to the ion masses of carbohydrate marker ions HexNAc^+^ (m/z 204.08 Da) and Hex-HexNAc^+^ (m/z 366.13 Da) are shown. Predicated number of glycosylated peptides containing these sugar markers are shown in Supplementary Figure S5.

**Supplementary Figure S5.** Distribution of probable glycosylated κA related conotoxins in the injected defensive and predatory venom of *C. striatus* (specimen D). Comparison of the glycosylated κA-conotoxin related mass units against the total conotoxin related mass units detected in reconstructed LC/MS/MS chromatograms of injected defensive and predatory venoms. κA-conotoxin related mass units were found by searching sugar ions HexNAc+ (204 Da) and Hex-HexNAc+ (366 Da). Hi and Low energy LC-ESI-MS runs (data not shown) revealed that most of the glycan moieties appeared as oligomers that could not be precisely characterized. All major peaks eluting between 30–45 min for the injected venoms were identified to have glycan moieties. By searching the presence of a carbohydrate marker ions HexNAc^+^ (m/z 204.08 Da) and Hex-HexNAc^+^ (m/z 366.13 Da) in the MS/MS sprectra we have found up to 466 peptides that were most likely κA-conotoxins based on their typical mass range (glycosylated mass range 4000–4900 Da), retention time (30–45 min) (most possibly κA-conotoxins as these are the only conotoxins found to have glycan groups and common occurrence in cone snails from the *Pinoconus* clade. Further clarification of glycosylated conotoxins is confounded by their sequence length (>38 amino acids), the diversity of possible PTMs, and likely heterogeneity of glycosylation. For these reasons, transcriptomic sequences for the κA-conotoxins could not be confidently matched to the MS-identified masses.

**Supplementary Figure S6.** Visualisation of the venom distribution pattern across eight venom duct sections and their correlation to the injected predatory and defensive venoms obtained from *C. striatus* specimen D. (a) Quantitative analysis of the major peptides found in the LC/MS profiles of the dissected venoms (venoms extracted from 8 duct segments). Both the retention time and the mass was considered when identifying the peptides across the duct sections and injected venoms. Peptides are ranked to the expression levels (relative to the maximum intensity of each sample) of section 1 (left) and 7 (right). (b) Mass profile comparisons were made between the LC/MS data obtained by injecting similar amount (1 μl) of the injected predatory and defensive venoms in comparison the distal duct section 2 and proximal duct section 7. Peptides are ranked by expression level (relative to the maximum intensity of each sample) of section 2 (left) and 7 (right).
