# Supplementary material for: Venom duct origins of prey capture and defensive conotoxins in piscivorous Conus striatus
Source: Sci Rep. 2021 Jun 24;11:13282. doi: 10.1038/s41598-021-91919-4 (PMC8225645; doi:10.1038/s41598-021-91919-4)

Annexe to Table 1.

**Integration of the transcriptome and proteomic data using ProteinPilot^TM^ tool.**

Underlined sequences are matched to 99% confidence to the proteomic data obtained from reduced alkylated and trypsin digested dissected and injected venom samples of *C. striatus.* MS/MS fragments obtained were used to map their presence in the proteome.

STR1_SI

ICCNPACGPKYSC


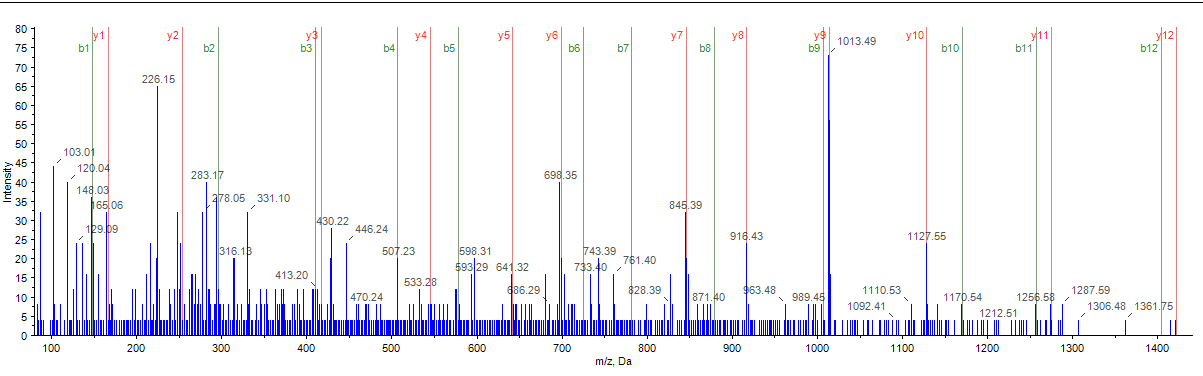


STR14_Sm1.2

NGCCRNPACESHRC


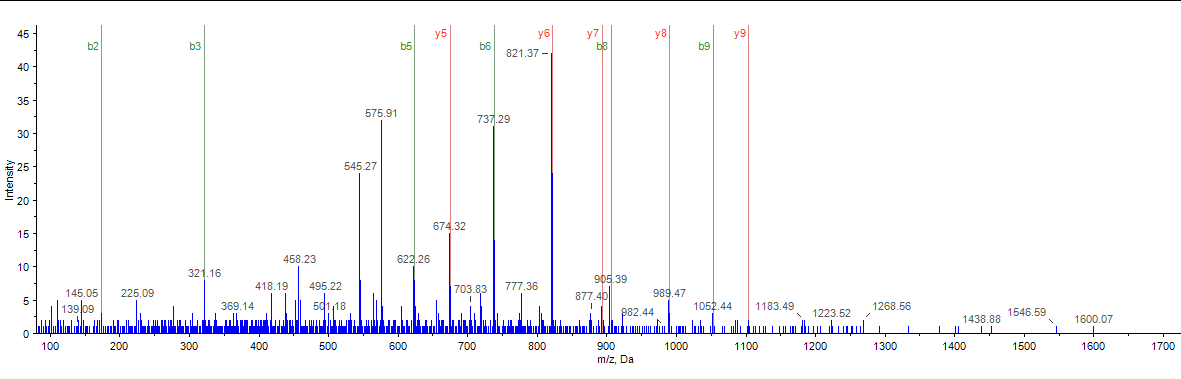


STR17_SII

GCCCNPACGPNYGCGTSCS


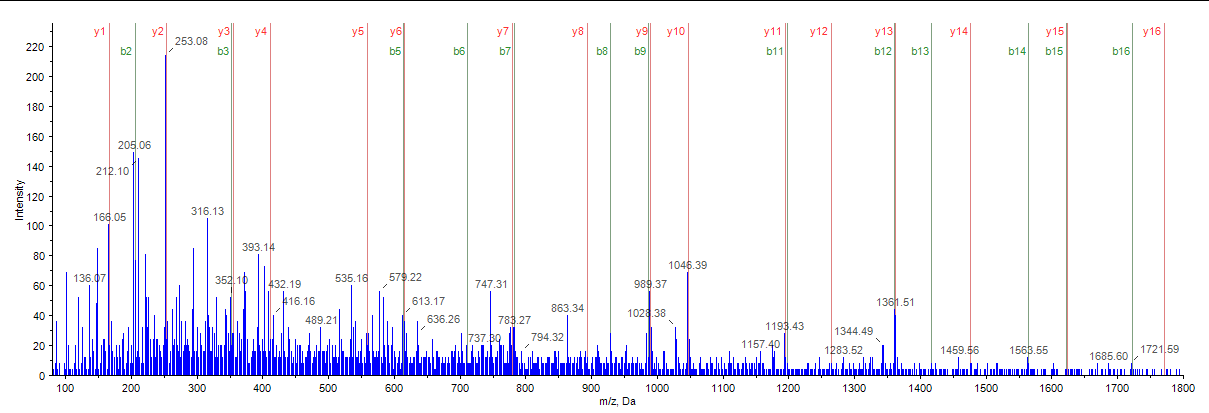


STR19

QKELVPSVITTCCGYDPGTMCPPCRCDNSCKPKPKK


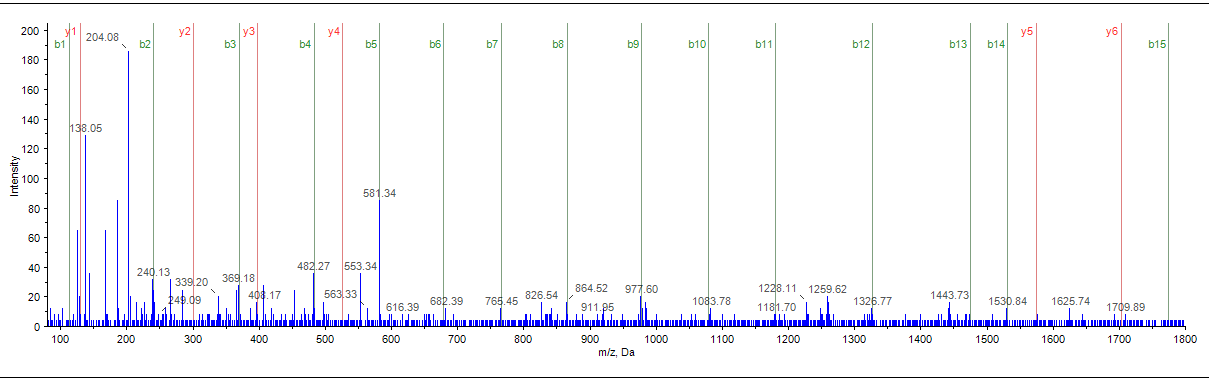


STR22

QKSLVPSVITTCCGYDPGTMCPPCRCTNSCPKKPKKP


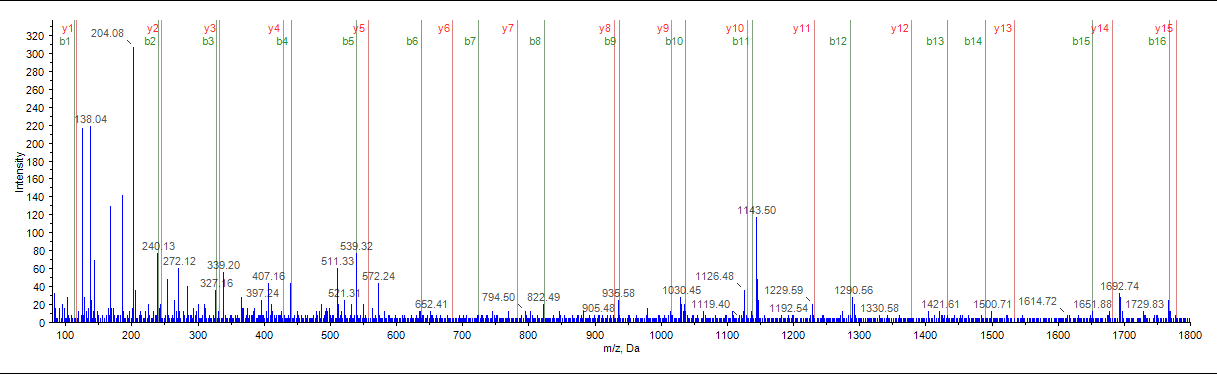


STR25

QKSLVPSVITTCCGYDPGTMCPPCRCTNSCKTKPKK


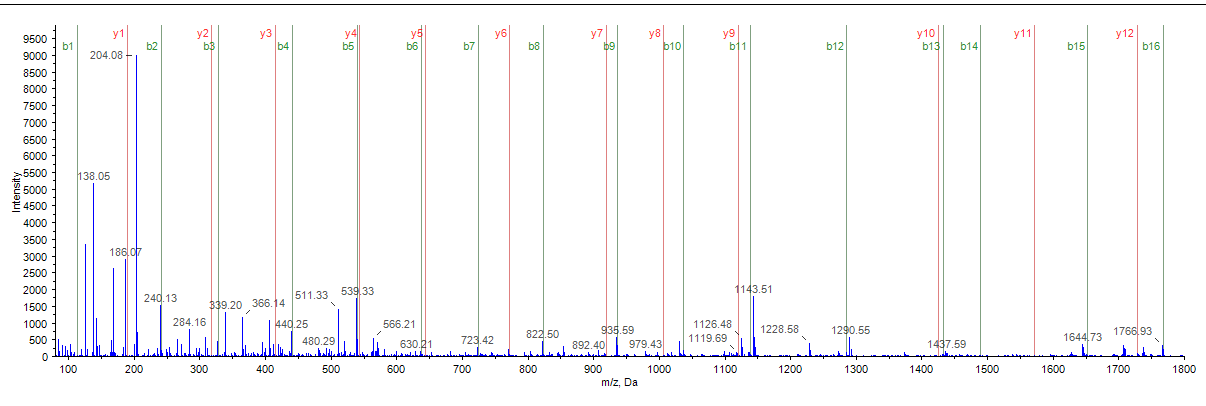


STR26

QKELVPSVITTCCGYDPGTMCPPCRCTNSCKTKPKK


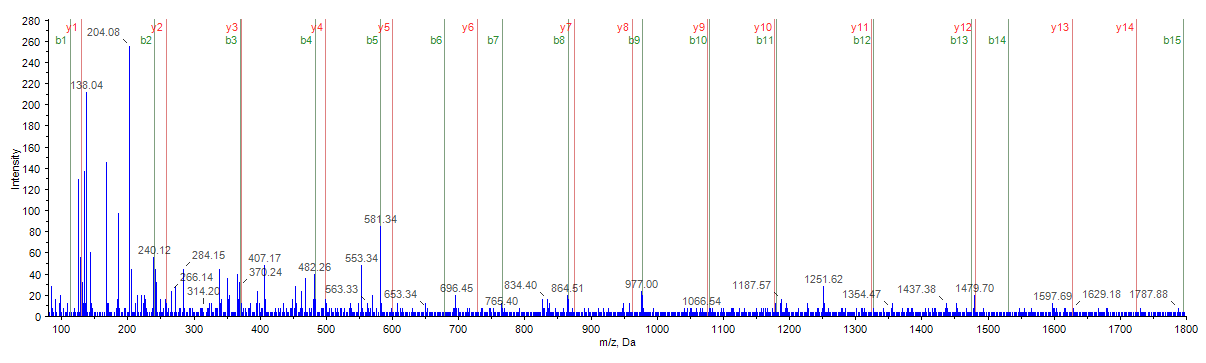


STR42_Con-ikot-ikot_SI

SGPADCCRMKECCTDRVNECLQRYSGREDKFVSFCYQEATVTCGSFNEIVGCCYGYQMCMIRVVKPNSLSGAHEACKTVSCGNPCA

SGPADCCR


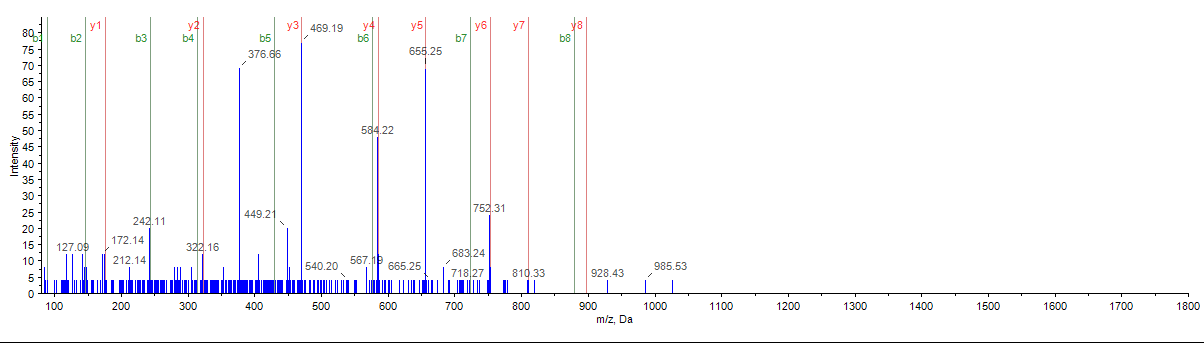


ECCTDRVNECLQR


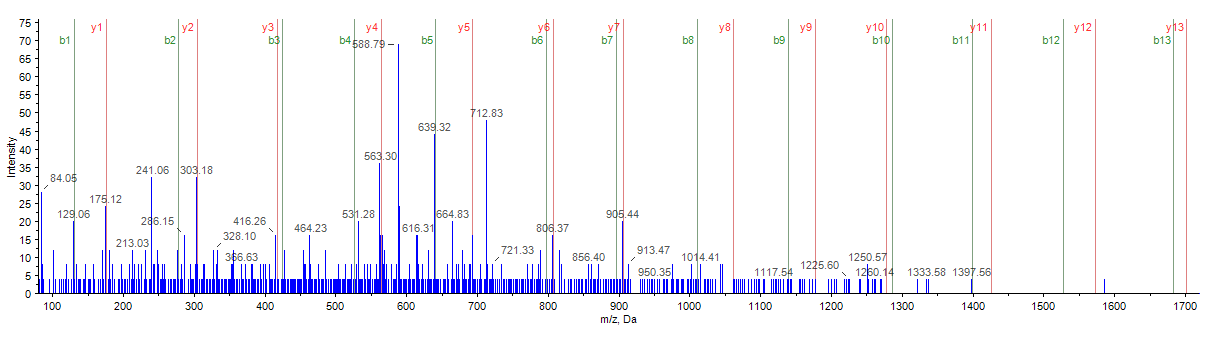


EDKFVSFCYQEATVTCGSFNEIVGCCYGYQMCMIRVVKPNSLSGAHEACK


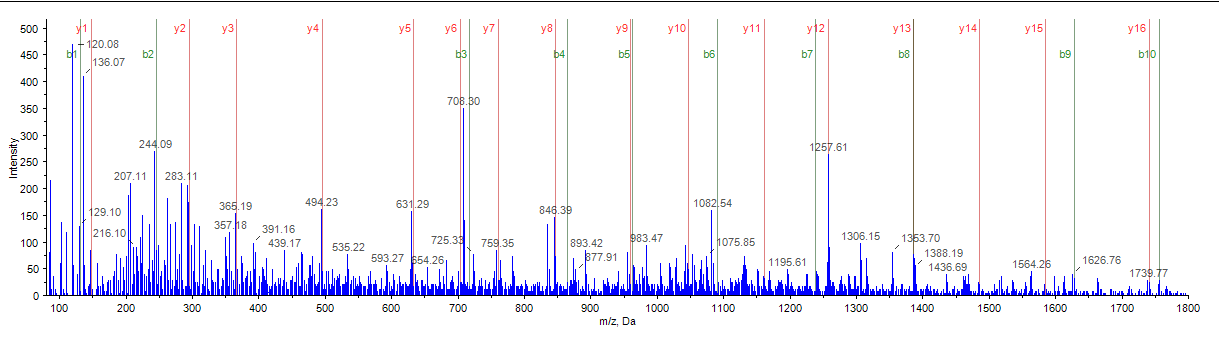


VVKPNSLSGAHEACKTVSCGNPCA


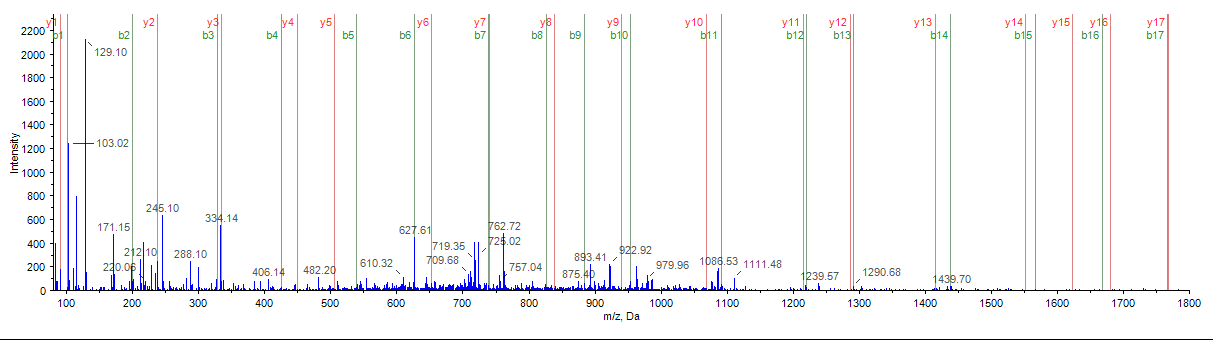


STR53

DRPSYCNLPADSGSGTKPEQRIYYNSAKKQCVTFTYNGKGGNGNNFSRTNDCRQTCQYPA

DRPSYCNLPADSGSGTKPEQR


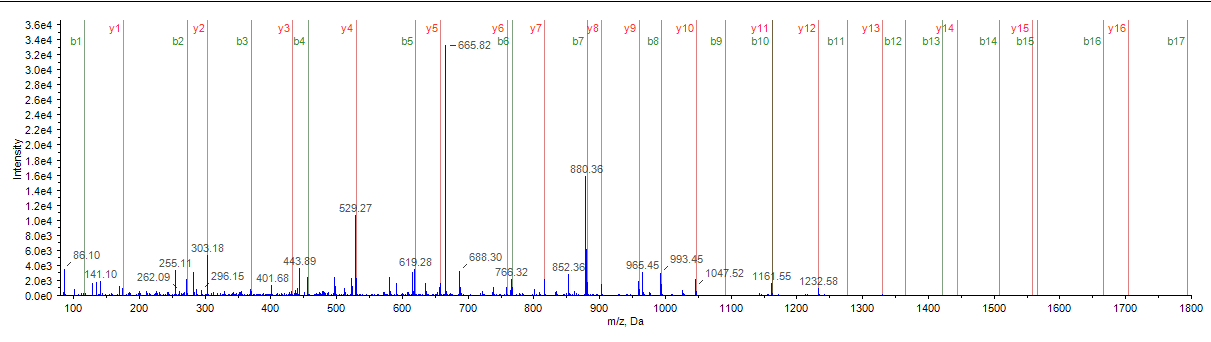


IYYNSAKK


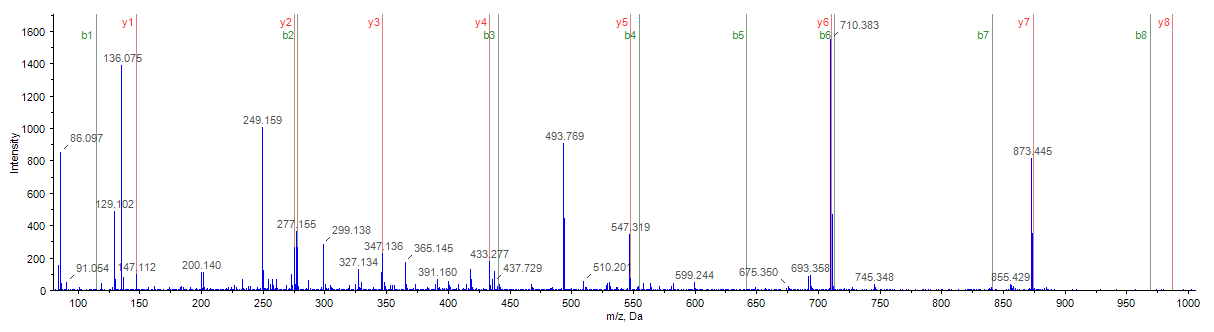


KQCVTFTYNGK


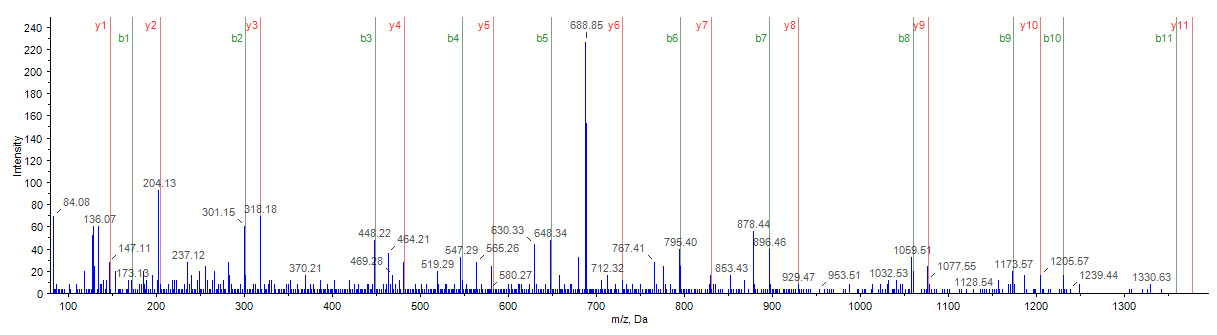


GGNGNNFSR


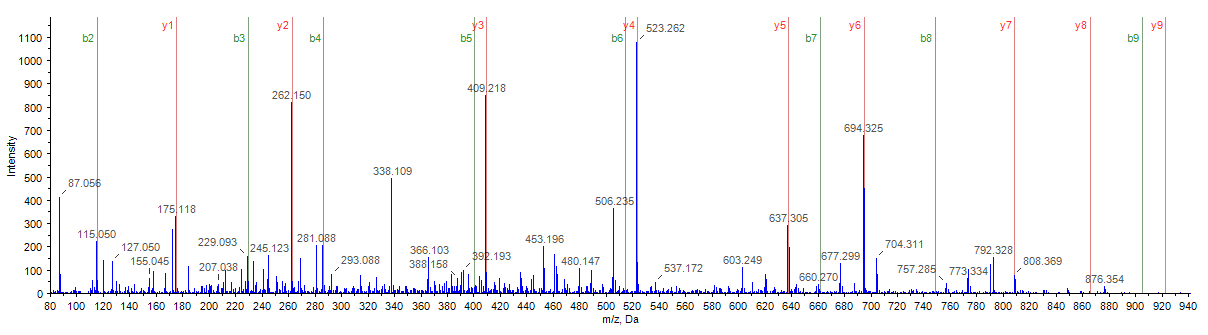


STR55_Conkunitzin-S2

ARPKDRPSYCNLPADSGSGTKPEQRIYYNSAKKQCVTFTYNGKGGNGNNFSRTNDCRQTCQYPV

ARPKDRPSYCNLPADSGSGTKPEQRIYYN


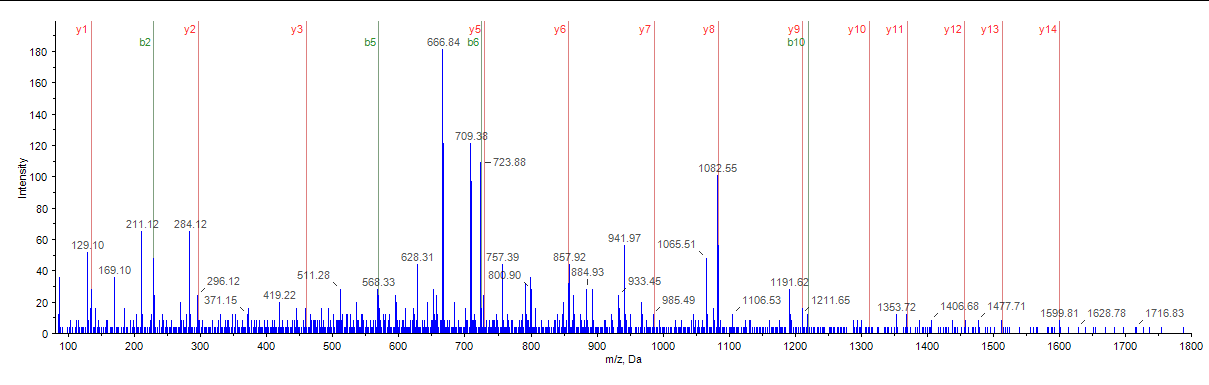


GNNFSRTNDCRQTCQYPV


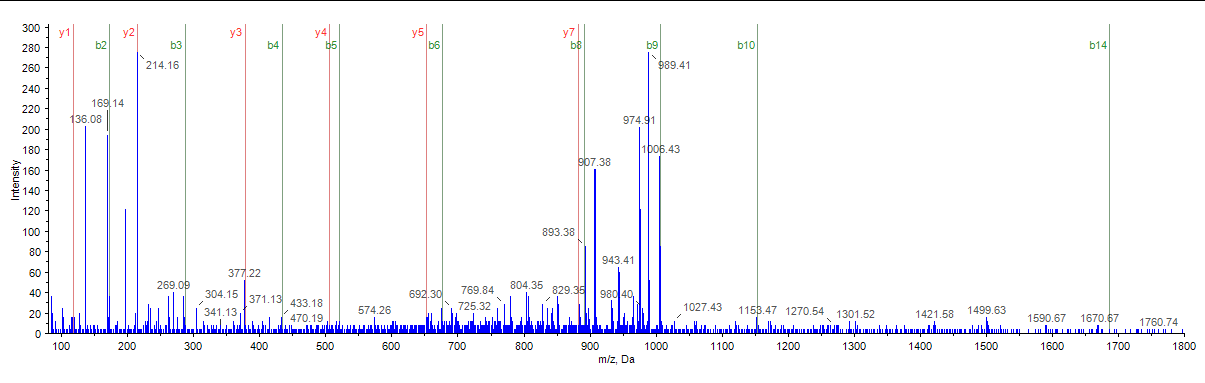


SAKKQCVTFTYNGKGGNGNNFSRTNDCRQTCQYPV


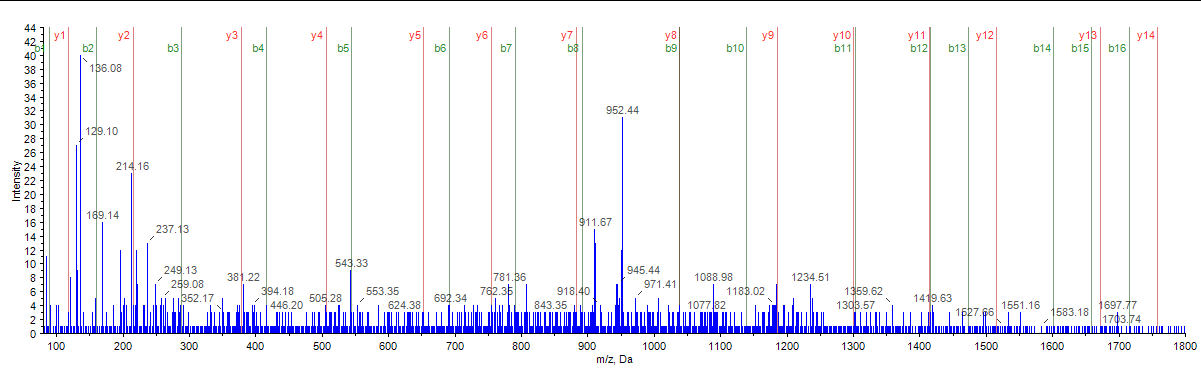


STR56_Conkunitzin-S1

KDRPSLCDLPADSGSGTKAEKRIYYNSARKQCLRFDYTGQGGNENNFRRTYDCQRTCLYT

KDRPSLCDLPADSGSGTKAE


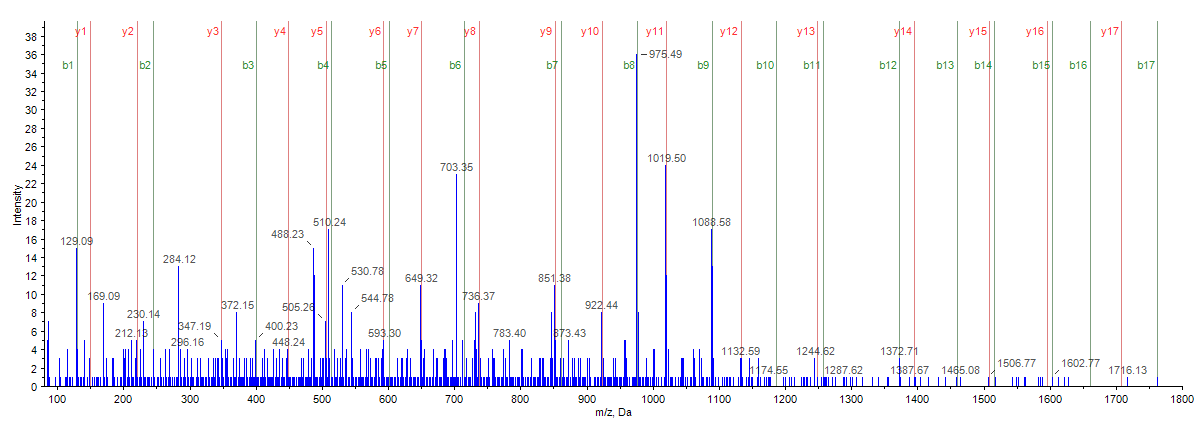


IYYNSARKQCL


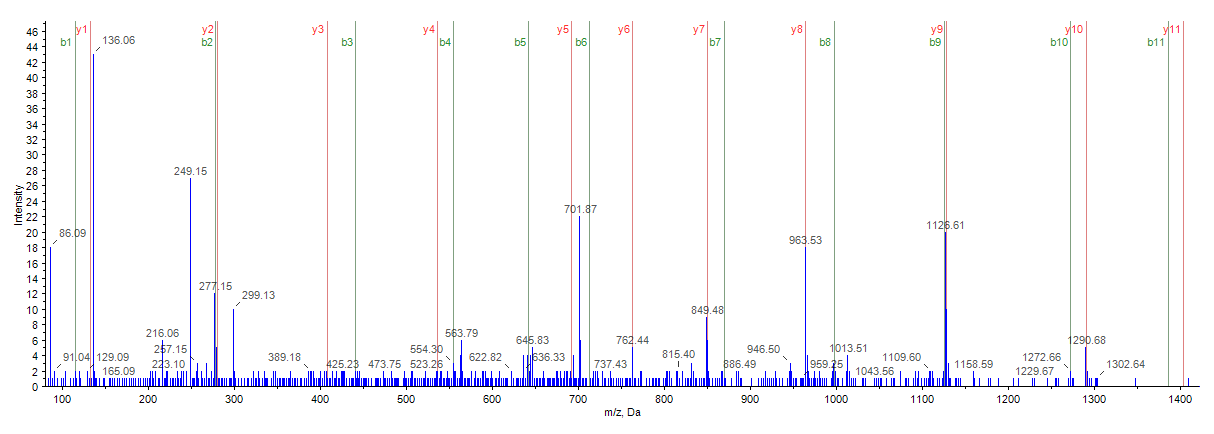


TYDCQRTCLYT


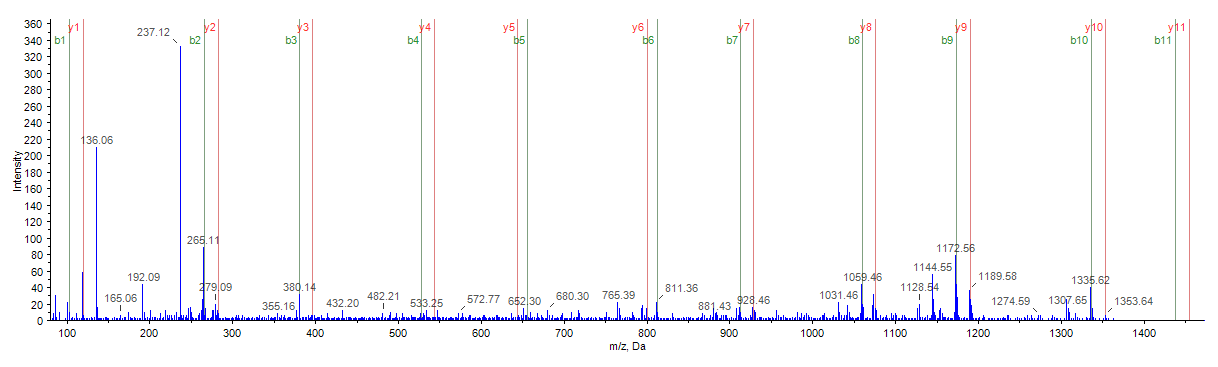


STR66

CIIRNCPRGGKRDVDETHLTMPCMCCSFRQCGAEYLLWSWRMGNGDRRSDQVH

CIIRNCPRGG


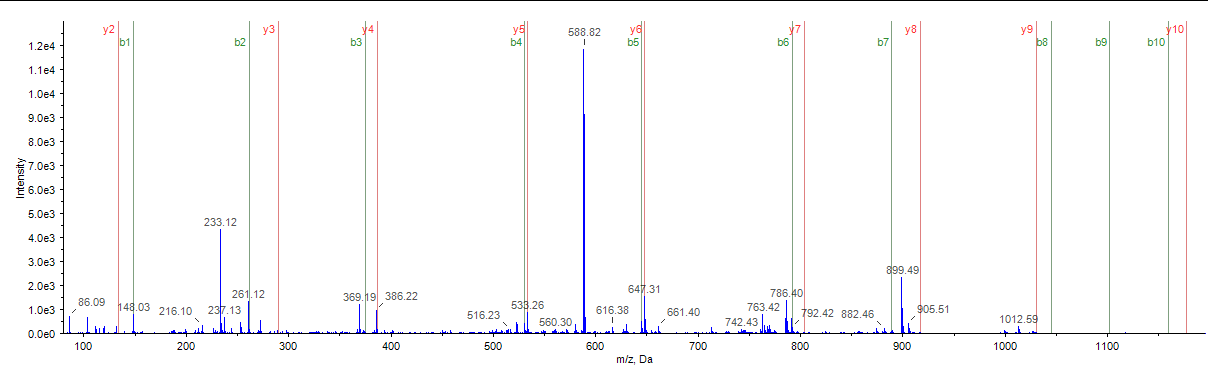


DVDETHLTMPCMCCSFRQCGAEYLLWSWRMGNGDRRSDQVH


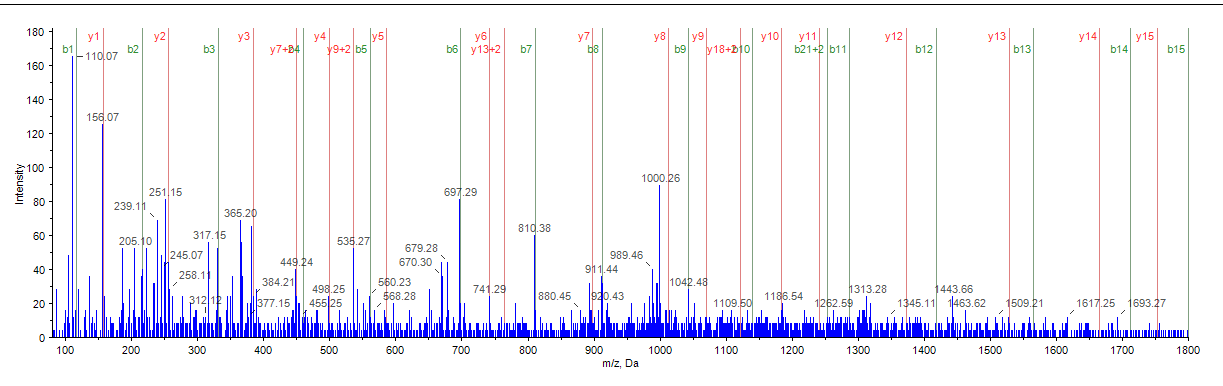


STR67

CIIRNCPRGGKRDVDETHLTMPCMCCSFRQCGAPYLLWSWRMGNGDRRSDQVH

CIIRNCPRGG


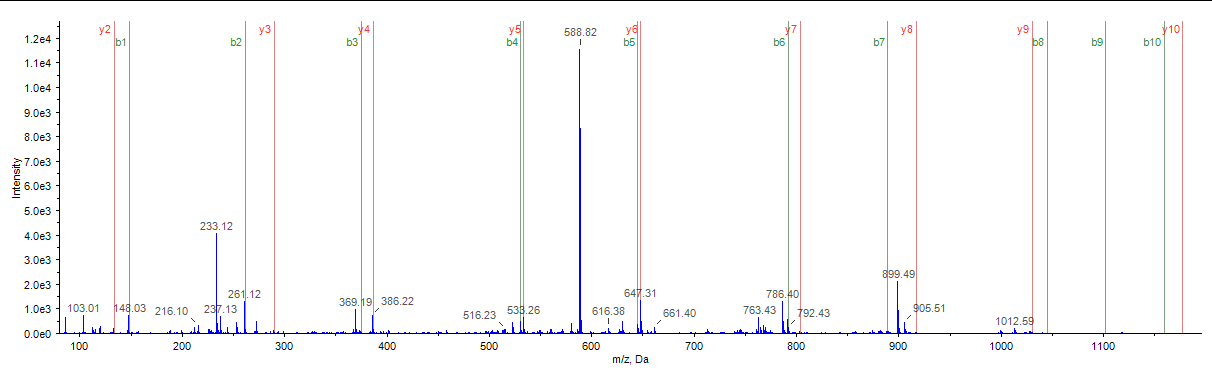


DVDETHLTMPCMC


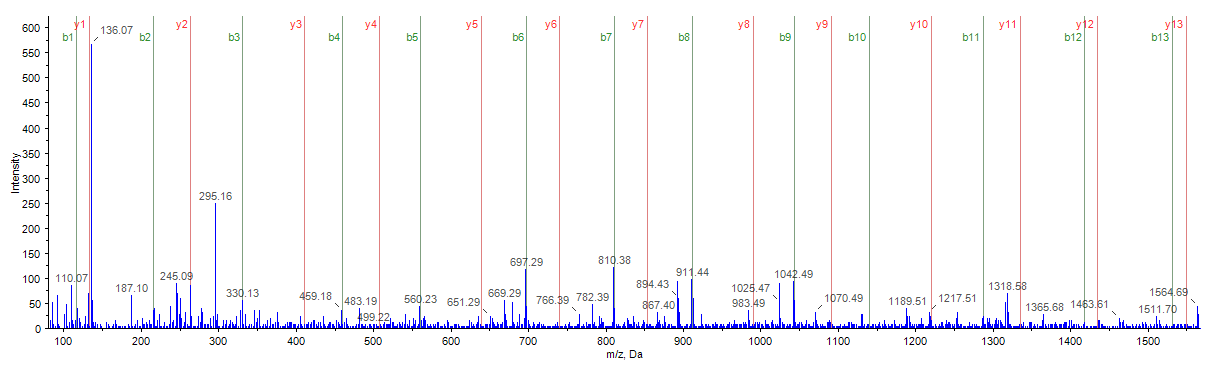


DETHLTMPCMCCSFRQCGAPYLLWSWRMGNGDRRSDQVH


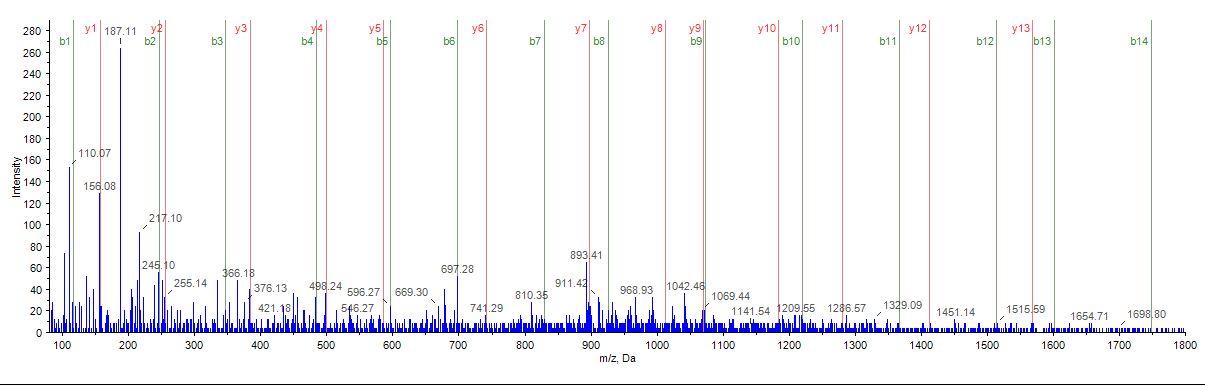


STR70

DCQRGCVGCGNRAGCCCGNKYCDKDNTCQEKPAKPST

GCVGCGNR


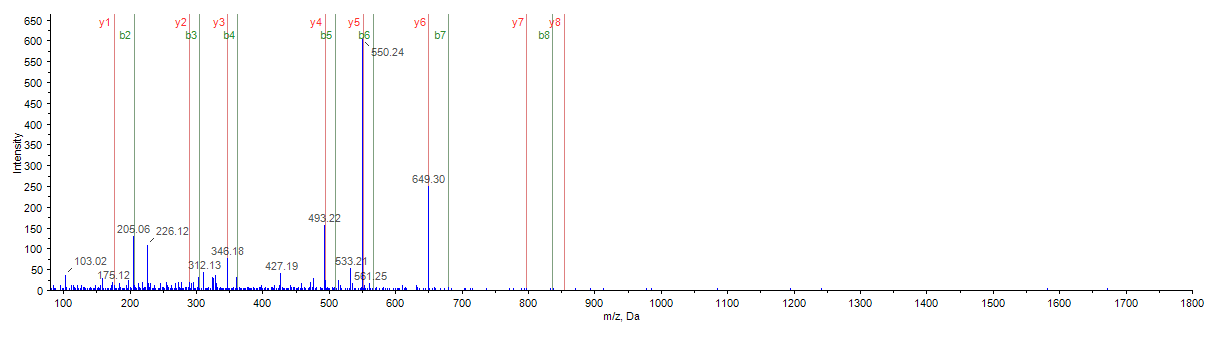


AGCCCGNK


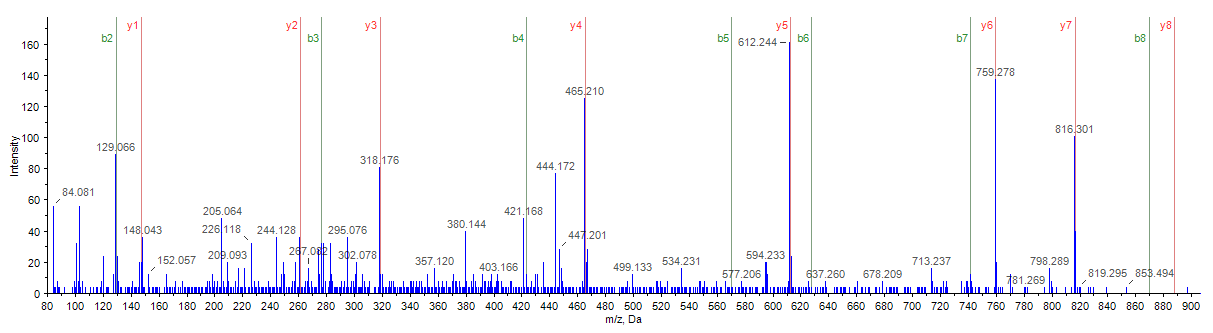


YCDKDNTCQEKPAKPS


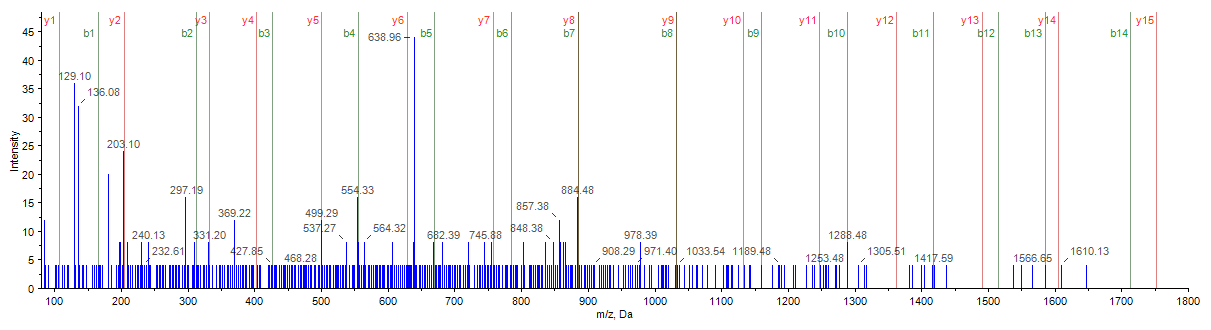


STR73

DSPQSECDGPRCPFICCFYEERKCGTRDCP

DSPQSECDGPR


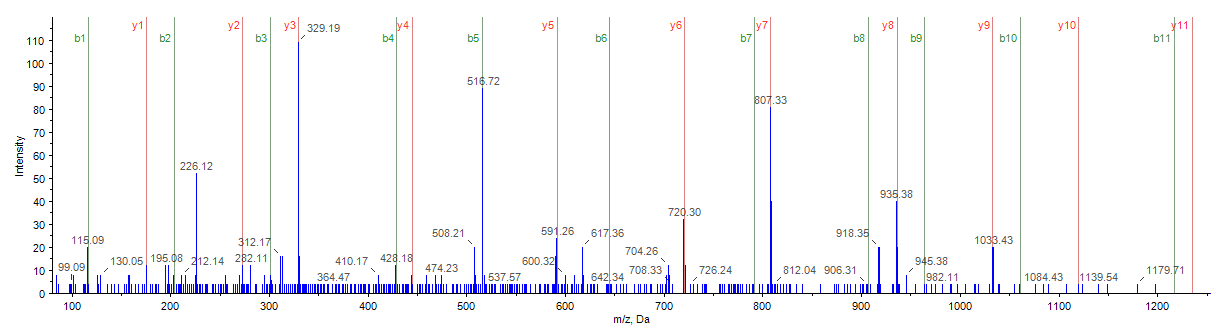


CPFICCFYEER


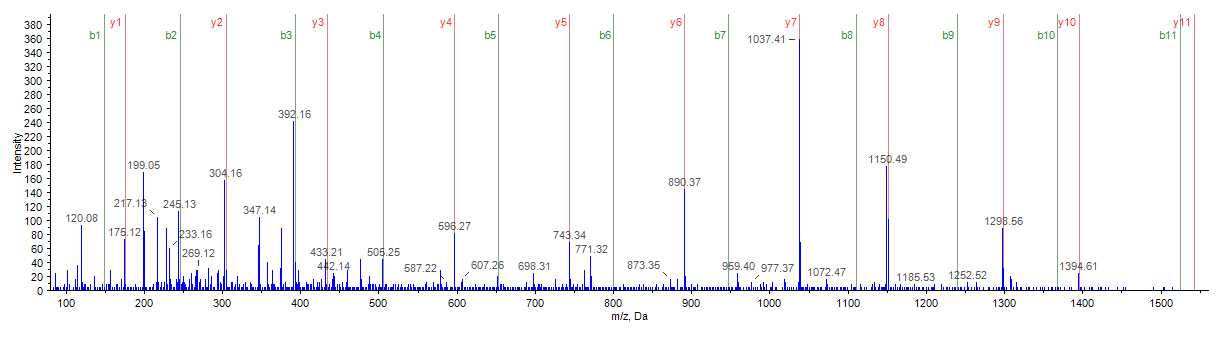


STR76

GTCSGVEQQCSNNADCCGELCCLSDKCGSPCMIRL


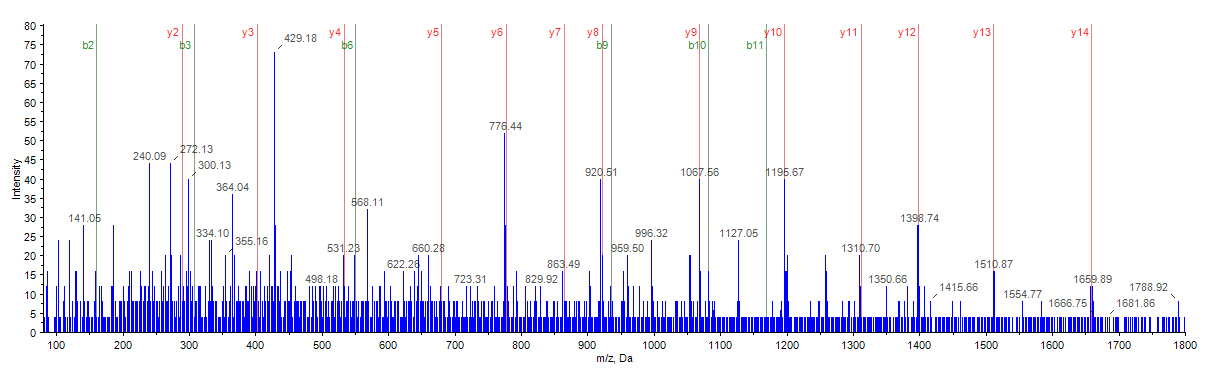


STR105

CCIAPMCRGPCKCCEEPGHP


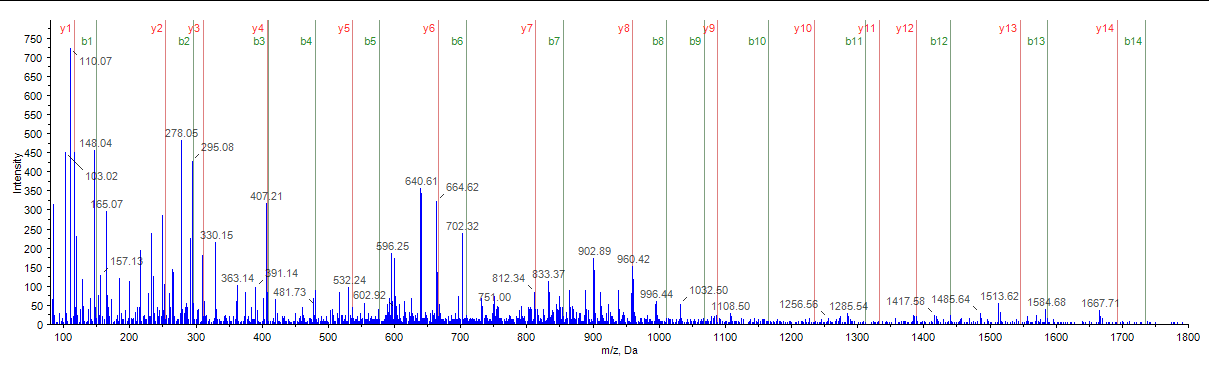


STR107_S3-S02

CCPARMCMAACSCCD


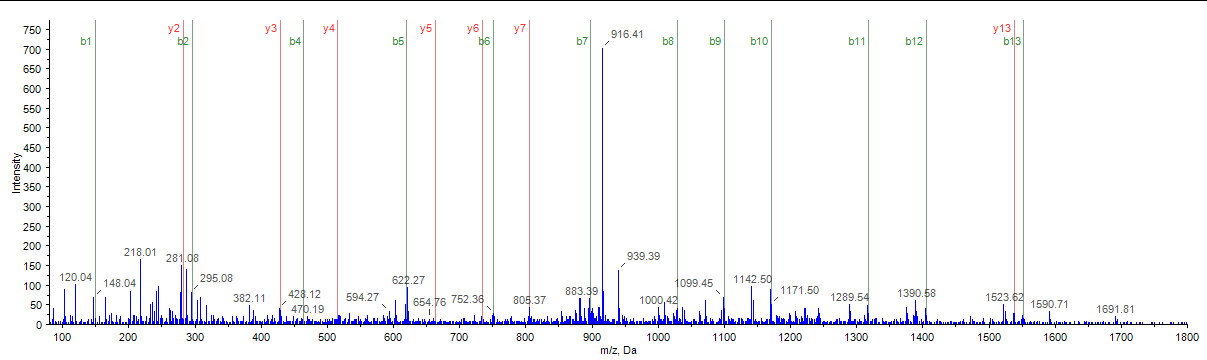


STR115_S3-G04

QKCCGEGSSCPKYFKNNFICGCC


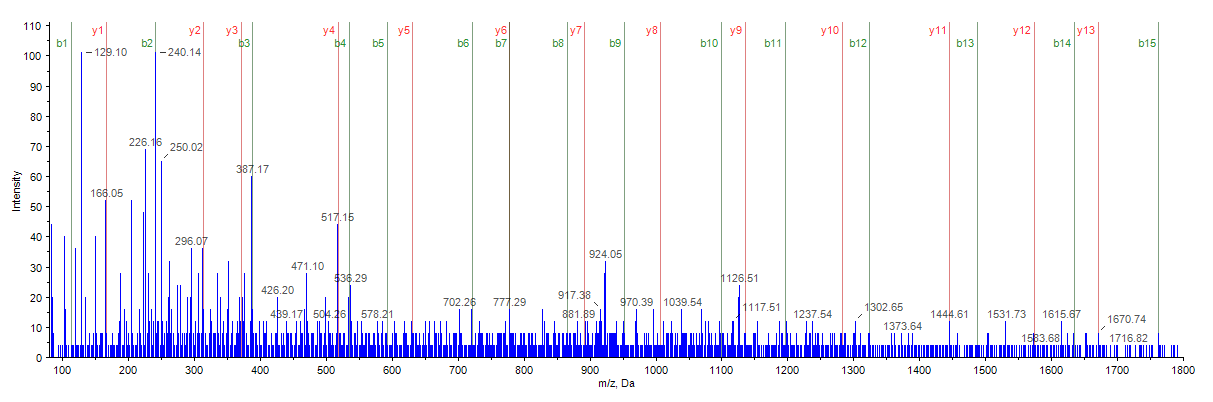


STR117_SIIIA

QNCCNGGCSSKWCRDHARCC

QNCCNGGCSSK


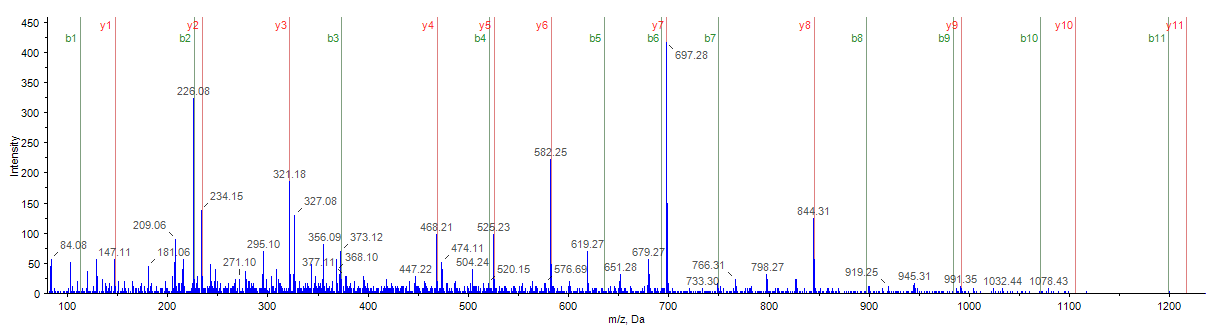


CRDHAR


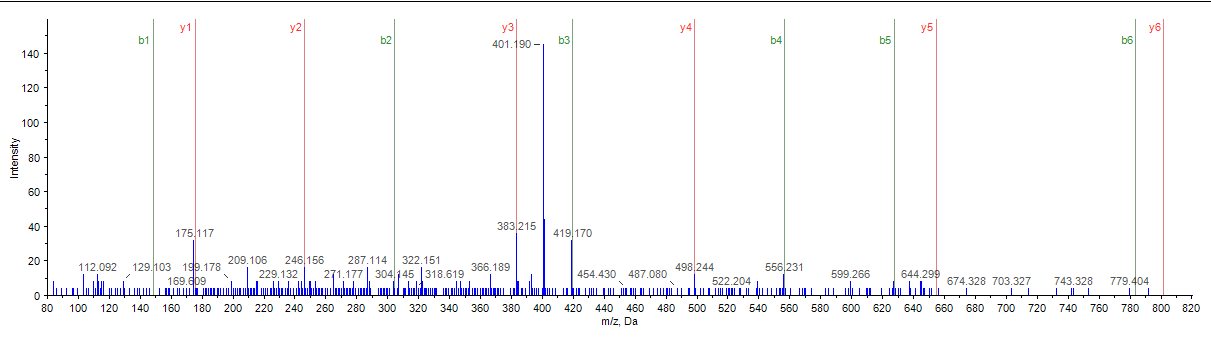


STR144_Conotoxin-3

CESYGKPCGIYNDCCNACDPAKKTCT


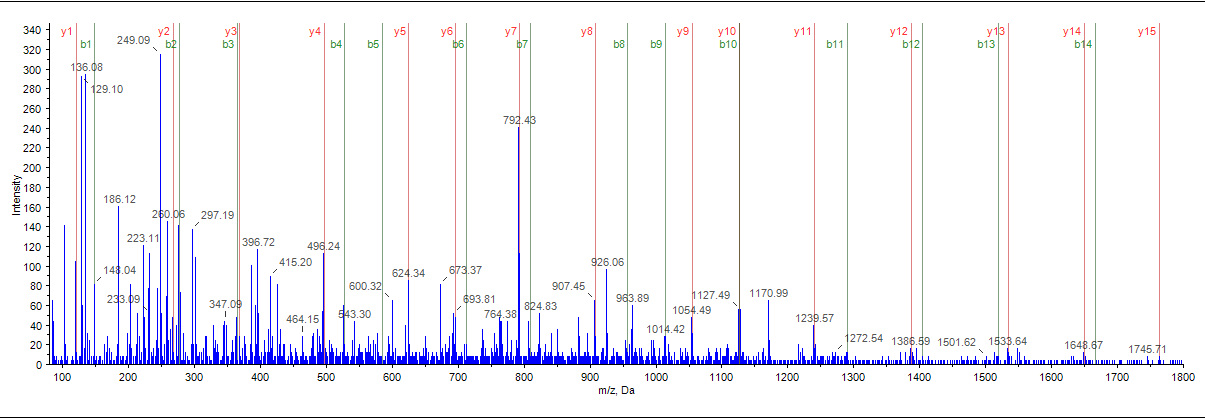


STR146_SO3

CKAAGKPCSRIAYNCCTGSCRSGKC


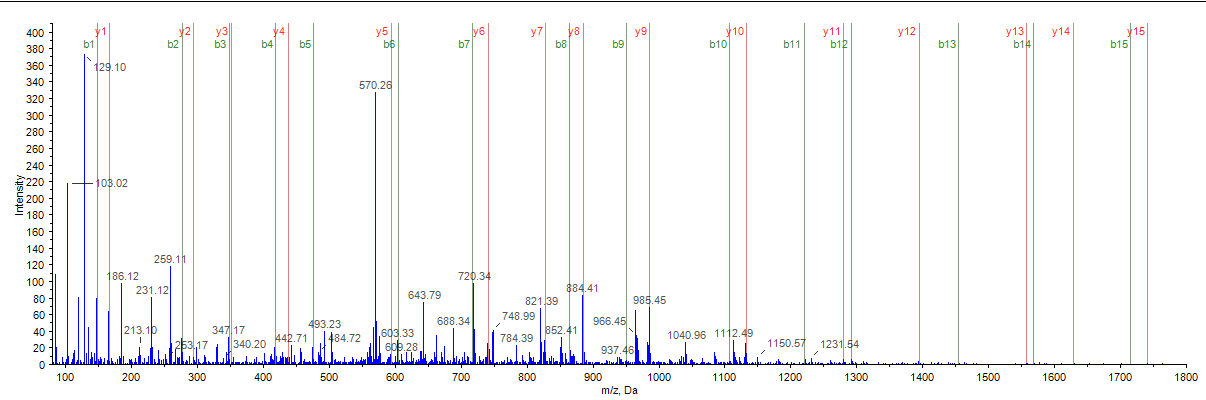


STR147_S6.1

CKAAGKSCSRIAYNCCTGSCRSGKC


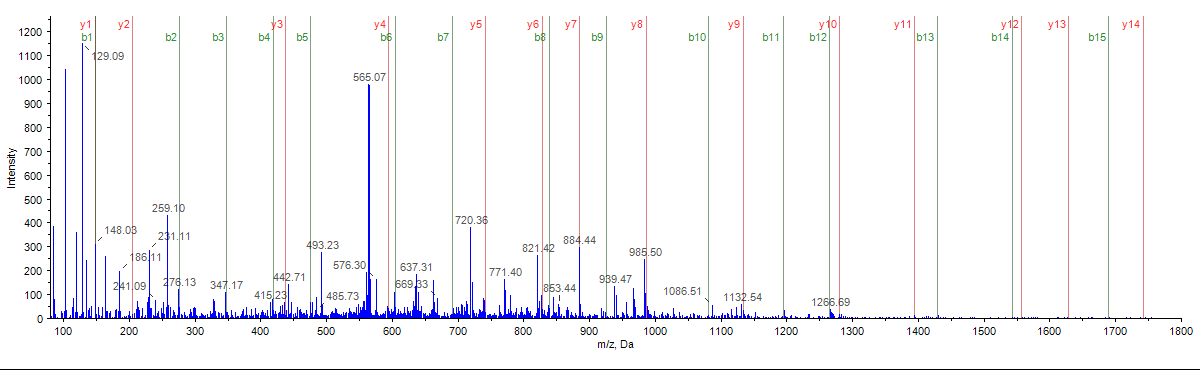


STR148_SVIB

CKLKGQSCRKTSYDCCSGSCGRSGKC


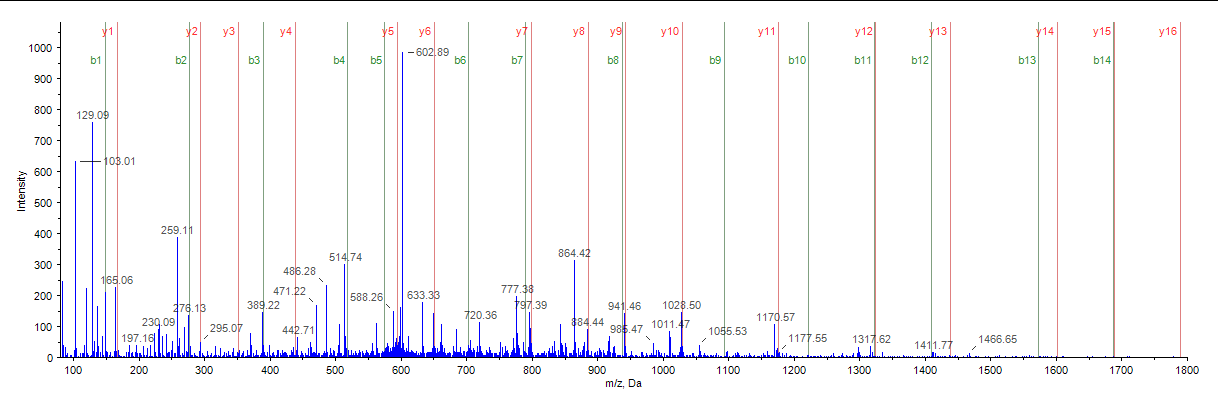


STR151_SVIA mutant 1

CRPSGSPCGVTSICCGRCYRGKCT


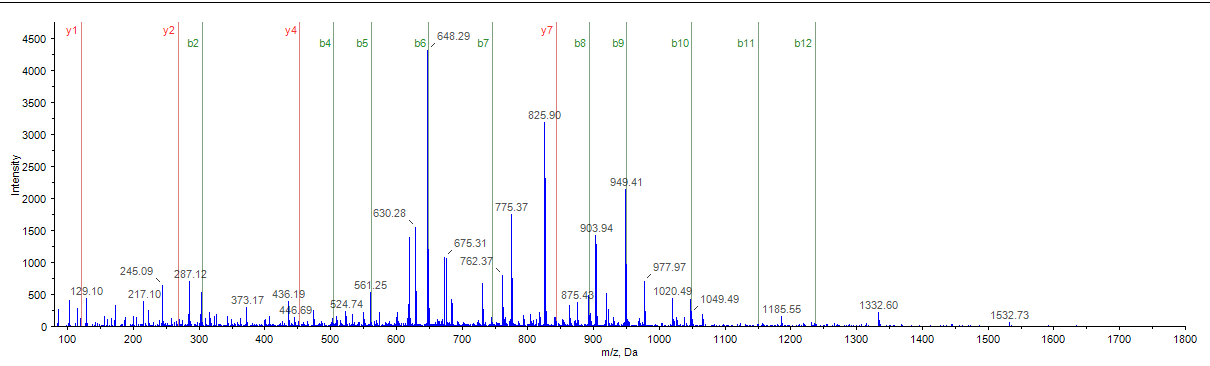


STR152_SVIA

CRSSGSPCGVTSICCGRCYRGKCT


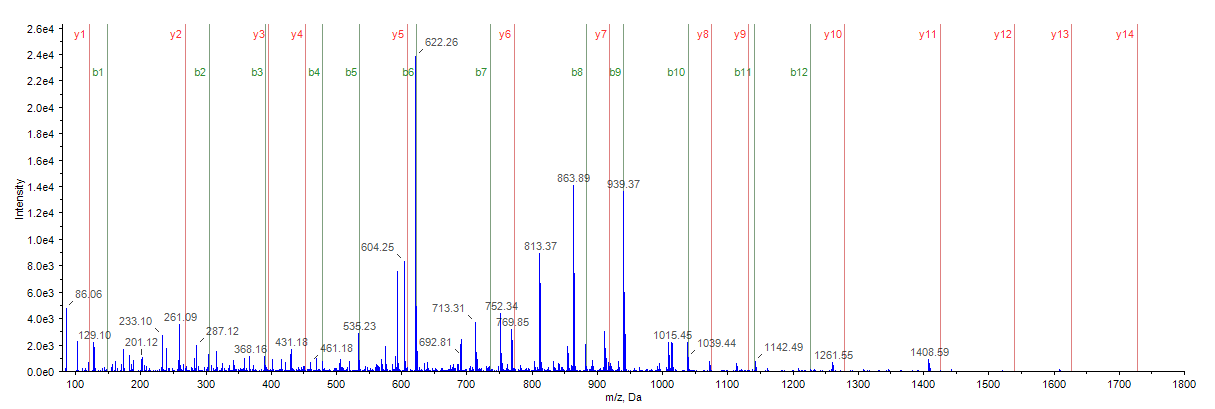


STR183_S6.8

DGCSNAGGFCGIHPGLCCSEICLVWCT


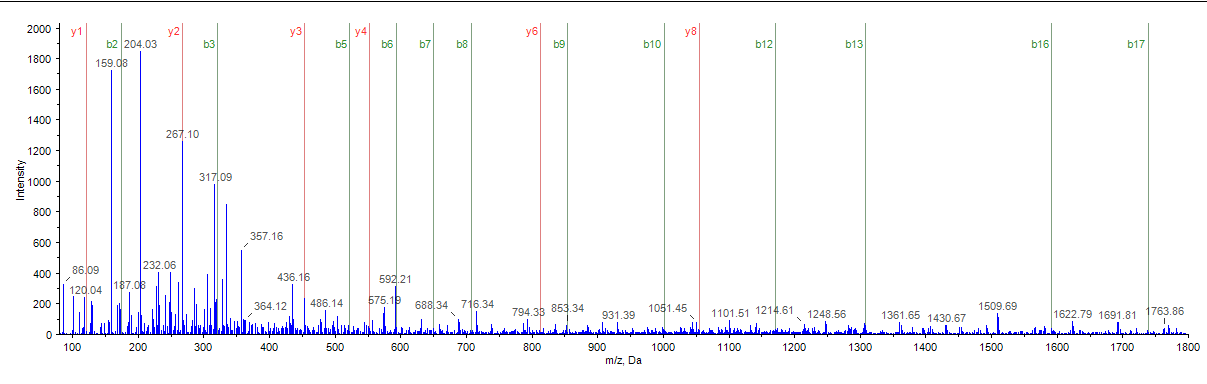


STR184_SVIE

DGCSSGGTFCGIHPGLCCSEFCFLWCITFID`


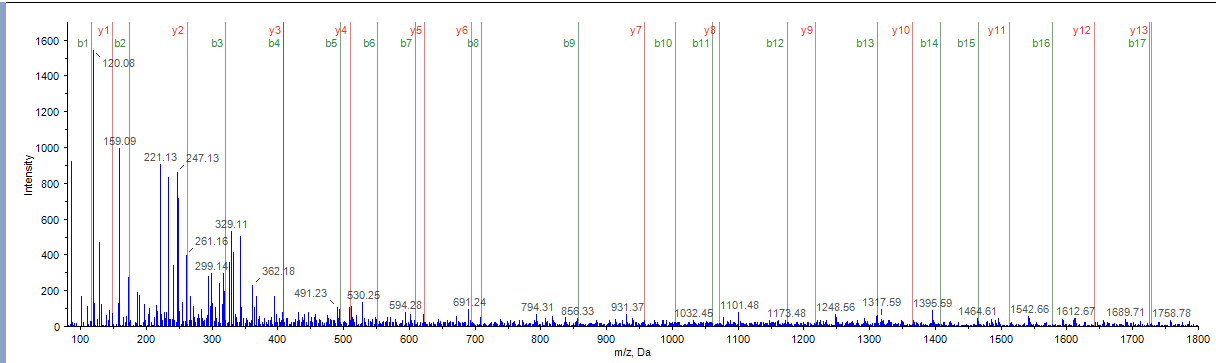


STR186

DKQEYHAVRKWSCVKRGDSCKTNICCAGLTCLRAHAINICLYLMPI


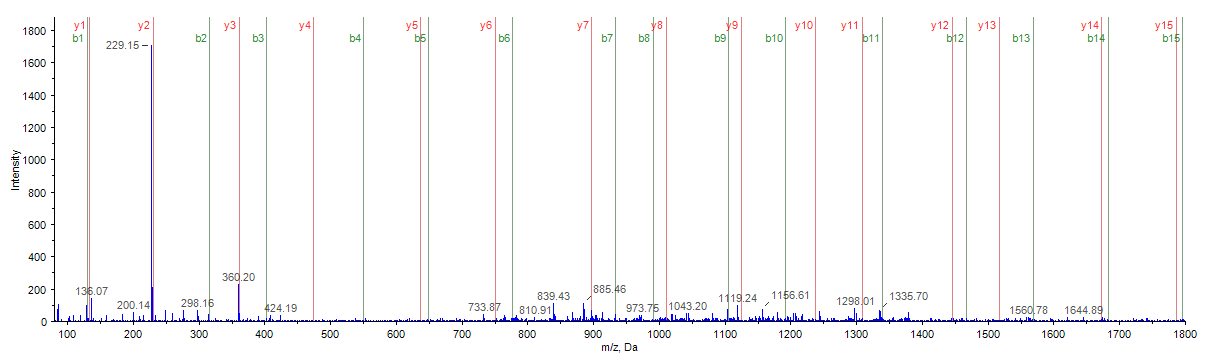


STR207

KSWSCVEHGDSCKTNICCAGLTCLRAHAINLCLYLMPM

KSWSCVEHGDSCK


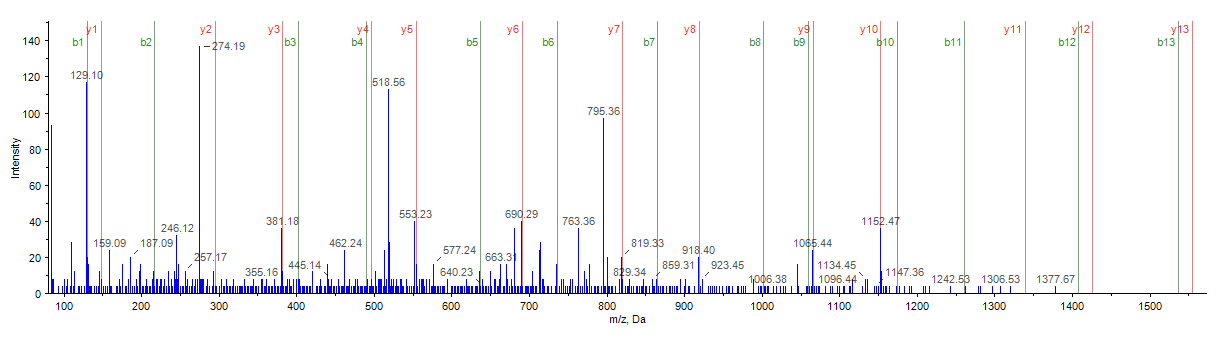


TNICCAGLTCLR


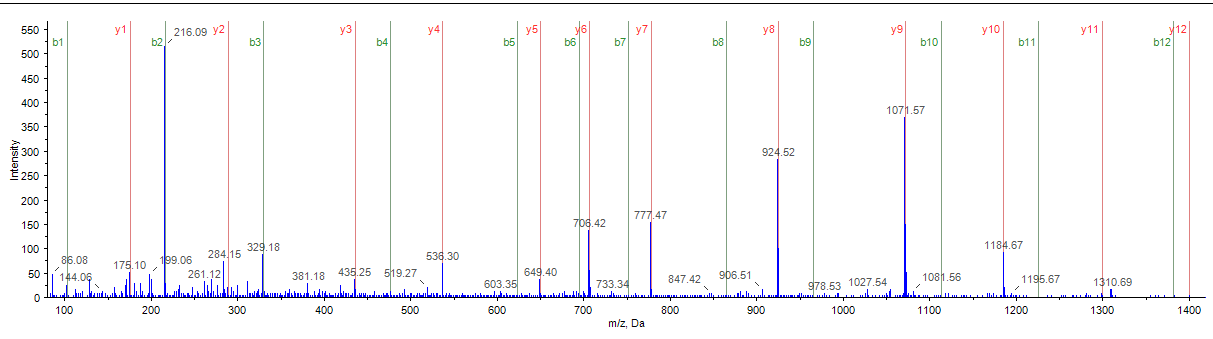


AHAINLCLYLMPM


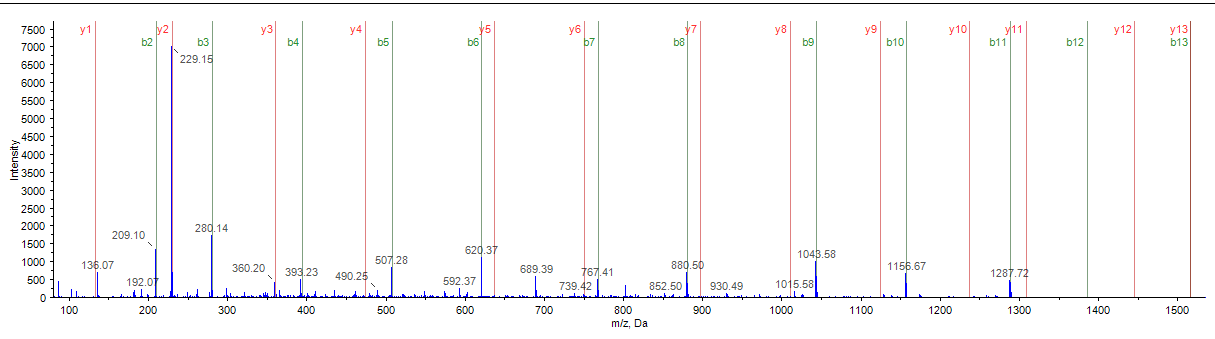


STR216-SO4

STTKVSKATDCIEAGNYCGPTVMKICCGFCSPYSKICMNYPKN


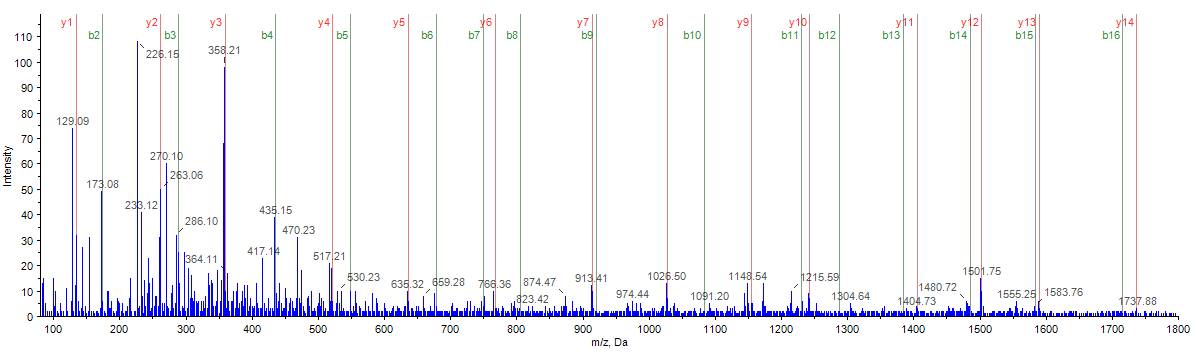


STR218_SO5

STTKVSKSTSCMEAGSYCGSTTRICCGYCAYFGKKCIDYPSN


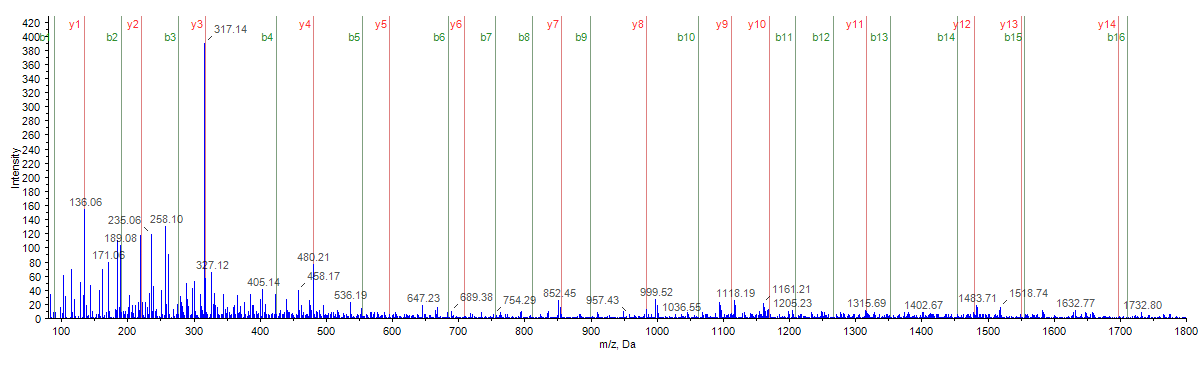


STR221

VRESDSCRKLGERCPSRPCCPRLRCGSGRAGGVCRHPYN

CPSRPCCPR


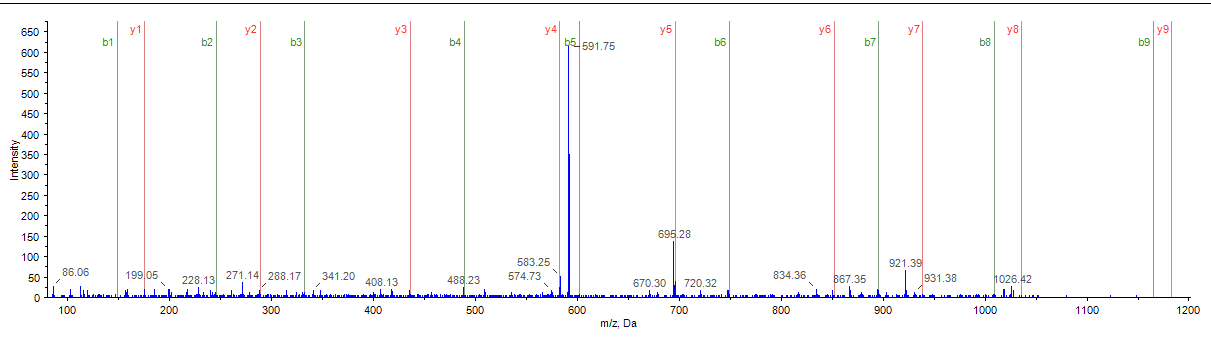


ESDSCRKLGE


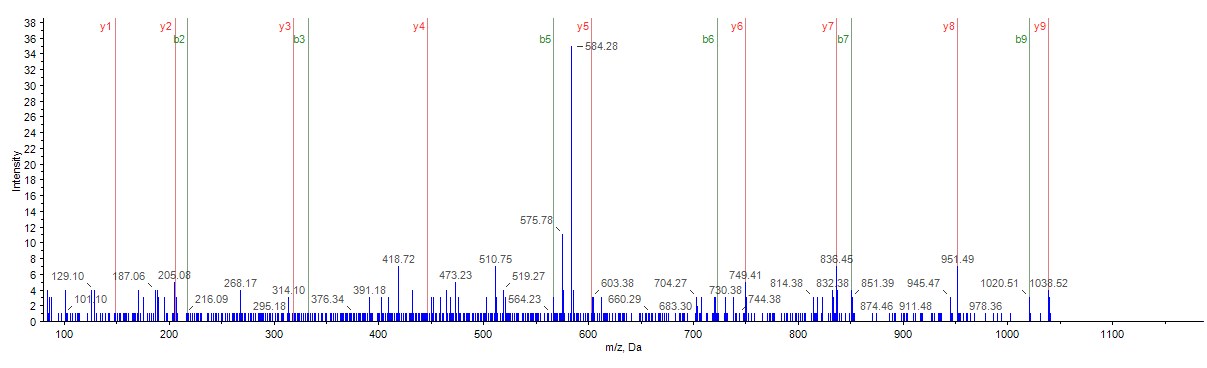


AGGVCRHPYN


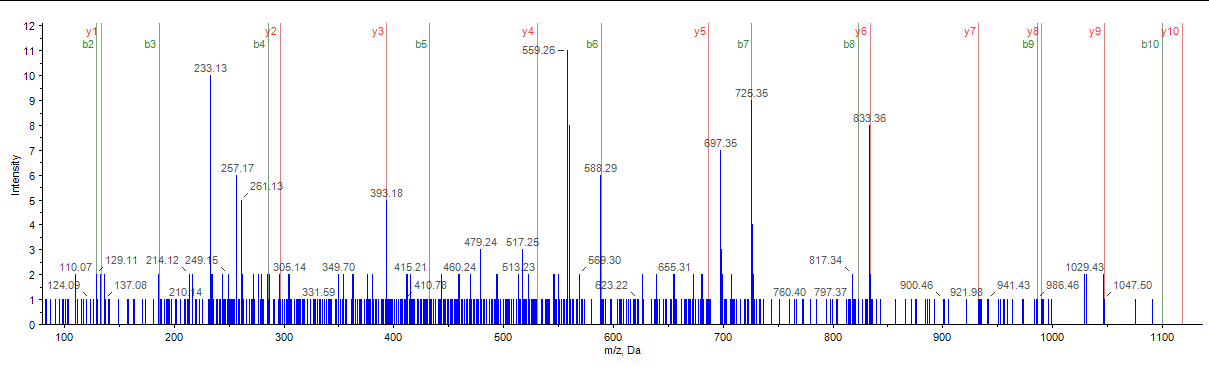


STR222

VRESEGCAGLGAPCRYRRCCRRLKCVGGHVGRACRYPANYYYYY

ESEGCAGLGAPCR


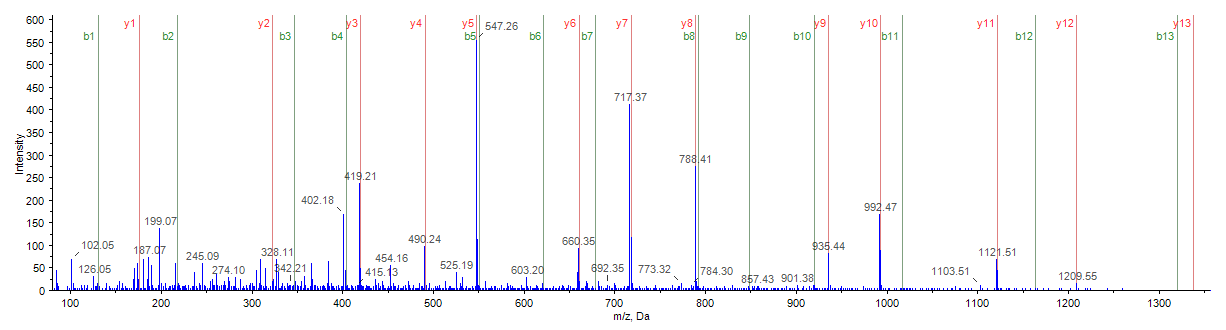


CVGGHVGR


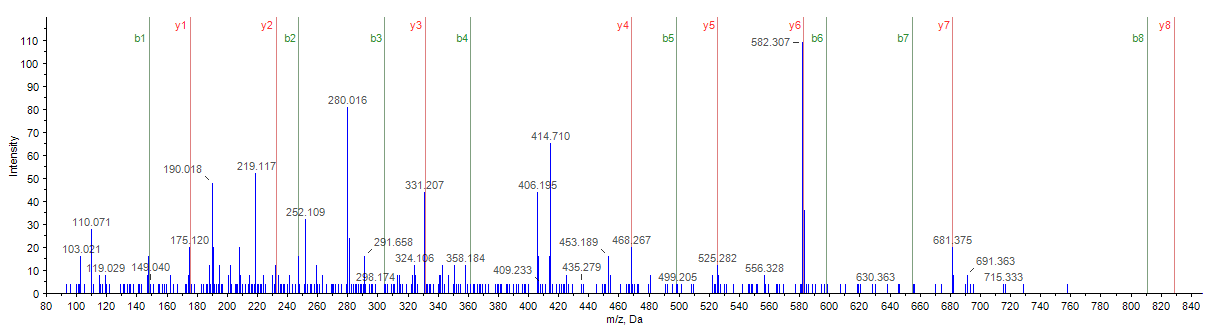


STR249

KNCAYAFDACTSDRQCCSGYCVGNVYCE


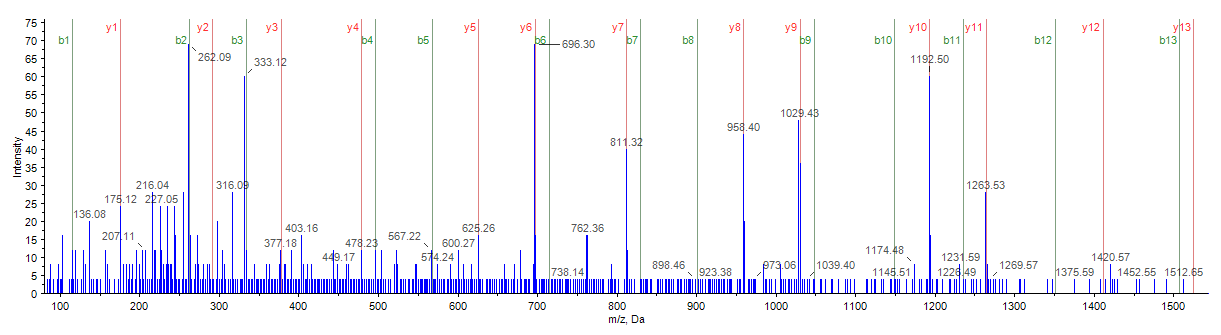


STR252

KSNAESWWEGECRTWNAPCSFTSQCCFGKCAHHRCIAW


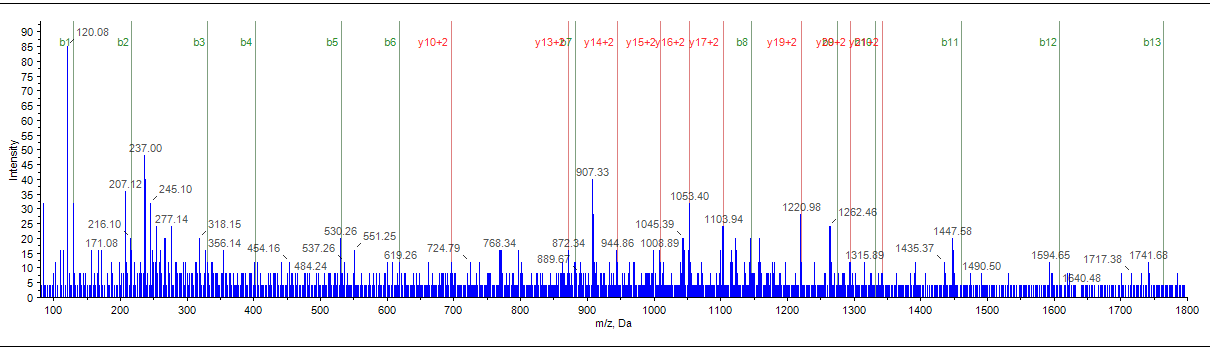


STR301

NTPDDGTCKSSSNCSTGQTCCKANAKNEKGFCTEDCWF

NTPDDGTCK


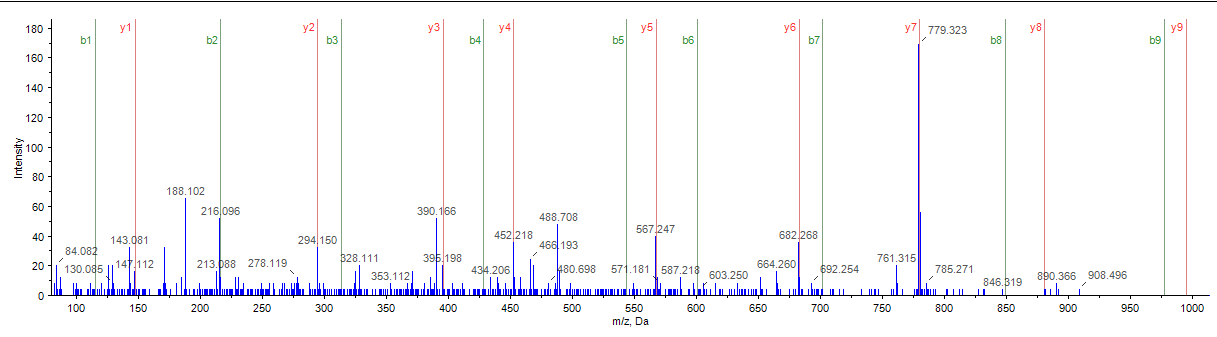


SSSNCSTGQTCCK


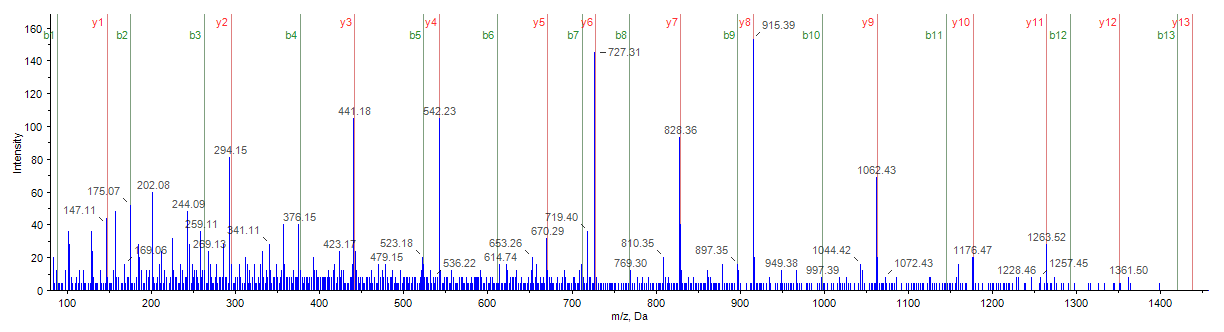


GFCTEDCWF


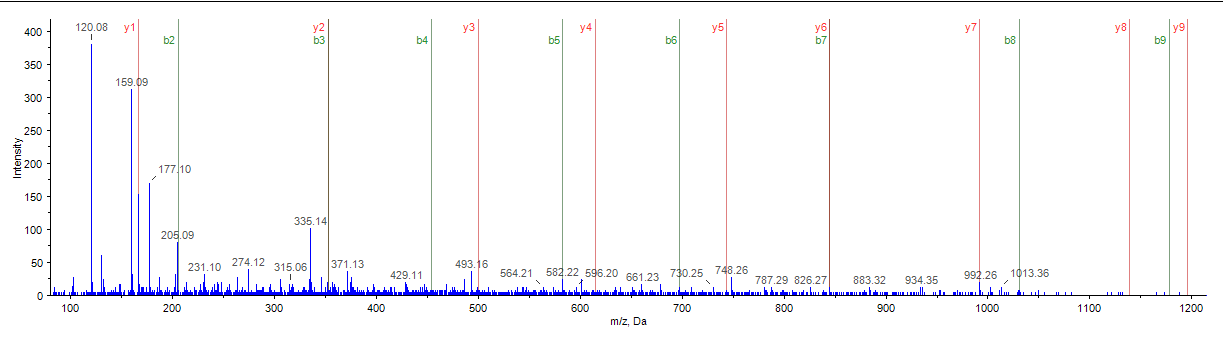


STR303

TADEACKEYCEERNKNCCGRTNGEPRCASMCF


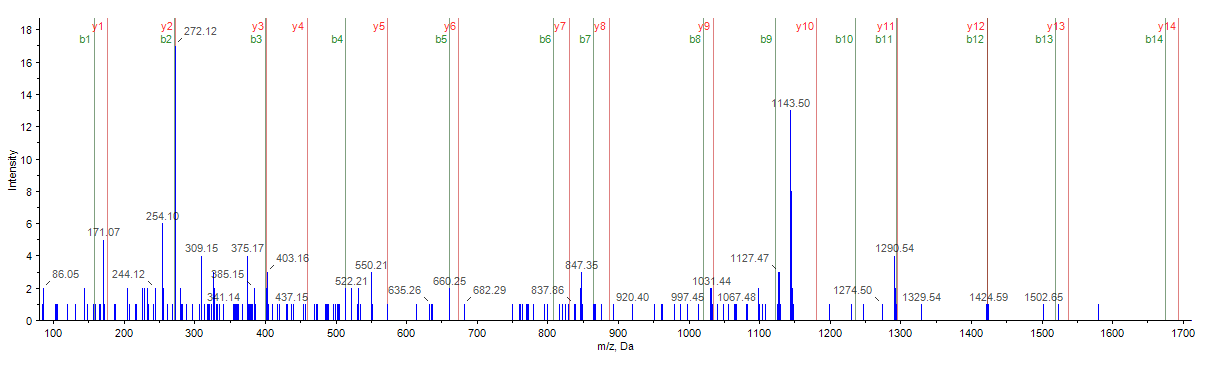


STR312

TVDEECKEYCEQRNKNCCGETNGEPVCAQACL


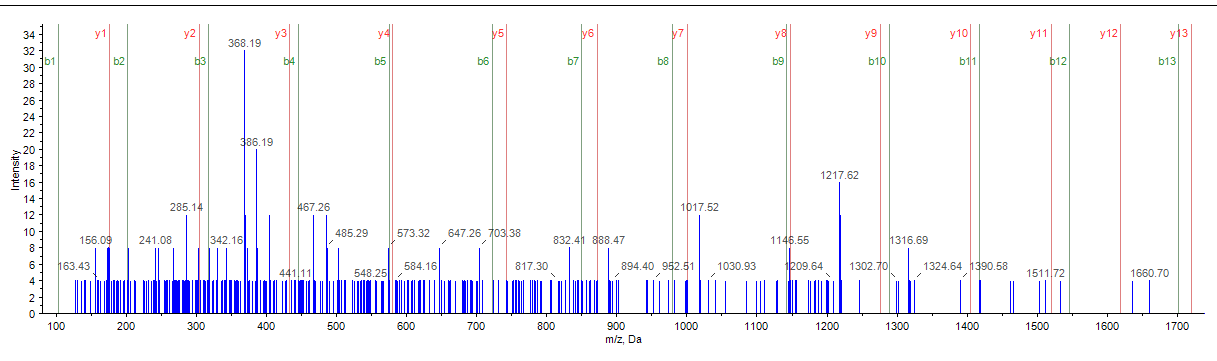


STR320

RVNCAGTLCQNGKCGGDCICRPANSTHQDCQPNDFD


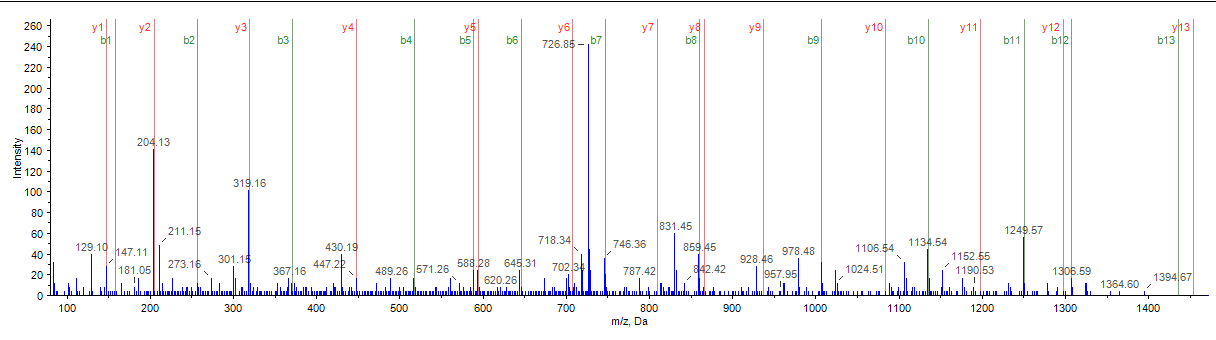


STR321

KSNAESWWEGECRTWNAPCSSTSQCCFGRCAHHRCIAW

KSNAESWWEGECR


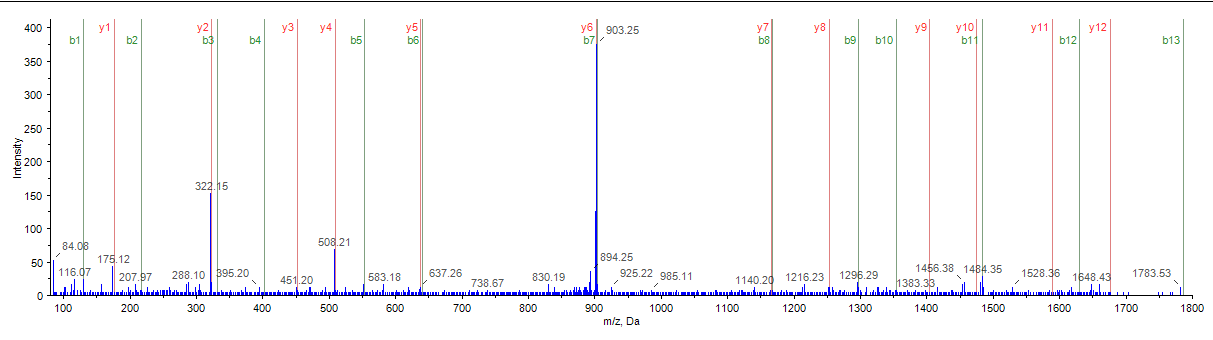


TWNAPCSSTSQCCFGRCAHHR


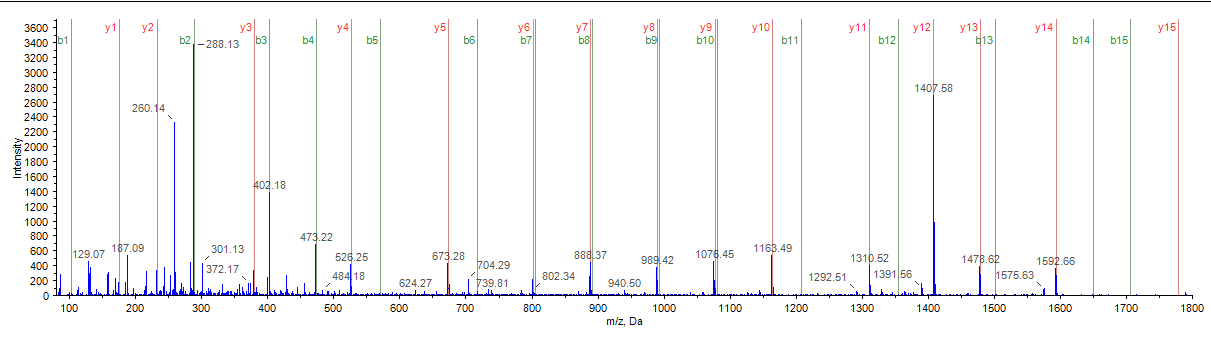


STR333

HCCPIDLPCCPL


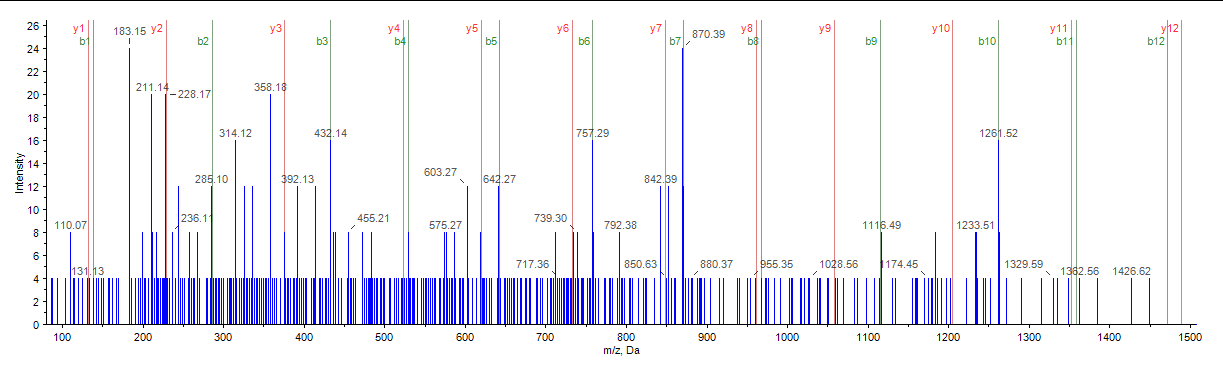


STR337

ISKSMGDVVGRTWWCPPEGELTHAGSATKQLLSSVWGLIGGVLRMLDQNRRH


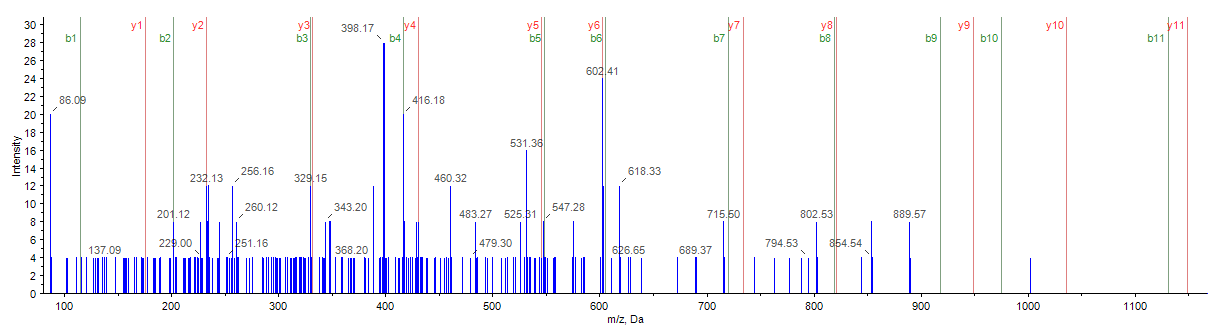

Supplement: Supplementary file 5 — Supplementary Dataset. [file 41598_2021_91919_MOESM5_ESM.docx]
